# Supplementary material for: Faecalibacterium prausnitzii Induces an Anti-inflammatory Response and a Metabolic Reprogramming in Human Monocytes
Source: Gastroenterology. 2026 Apr;170(4):704–20. doi: 10.1053/j.gastro.2025.10.003 (PMC13006548; doi:10.1053/j.gastro.2025.10.003)
Supplement: Supplementary Data [file mmc1.pdf]

Supplementary figure and table legends

Supplementary figure 1. (A) Cytokine concentrations and anti-inflammatory ratios for IL-10 and IL-23 obtained by ELISA analysis (corrected by LDH) in the supernatant of $CD14^+$ monocytes isolated from PBMCs and stimulated in the same conditions as in Fig.1 and 2 (N=8). **P*<.05, ***P*<.01 and ****P*<.001 as determined by mixed-effects analysis and Dunnett's multiple comparisons test. (B) Gating strategy for flow cytometry analysis of the different immune cell populations in PBMCs.

Supplementary figure 2. (A) Gating strategy for flow cytometry analysis of monocytes in PBMCs. (B) FACS plot representing CD14 and CD16 expression in PBMCs stimulated by different doses of *F. prausnitzii* EXL01 strain (Fp1, Fp10, Fp100) or LPS. (C) Percentage of classical ($CD14^+CD16^-$), intermediate ($CD14^+CD16^+$), non-classical ($CD14^-CD16^+$) and total $CD14^+$ monocytes among live stimulated PBMCs. (D) FACS plot representing the percentage of $CD14^+$ cells before and after isolation of $CD14^+$ cells from PBMCs using magnetic beads.

Supplementary figure 3. IL-10 concentrations measured by ELISA (corrected by LDH) in the supernatant of intestinal mucosa (ileum and colon) from non-inflamed controls (adenoma-carcinoma or ADK patients) and IBD patients showed separately, stimulated for 16 h with different doses of *F. prausnitzii* EXL01 strain (Fp1, Fp10, Fp100) or LPS, as performed previously. N=23 (IBD ileum N=6; IBD colon N=8; ADK ileum N=5; ADK colon N=4). Data are mean ± SEM.

Supplementary figure 4. (A) Gating strategy for flow cytometry analysis of IL-10^+ monocytes in the lamina propria immune cells isolated from intestinal mucosa. (B) Percentage of IL-10^+ cells among $CD14^+$ monocytes in the lamina propria immune cells isolated from intestinal mucosa (ileum and colon) from ADK and IBD patients showed separately, stimulated for 16 h with different doses of *F. prausnitzii*

EXL01 strain (Fp1, Fp10, Fp100) or LPS, as performed previously. N=9 (IBD ileum N=3; IBD colon N=2; ADK ileum N=1; ADK colon N=3). (C) Percentage of IL-10$^+$ cells among CD3$^+$ T and CD19$^+$ B cell populations in the lamina propria immune cells isolated from intestinal mucosa (ileum and colon) from IBD and ADK patients, stimulated for 16 h, as performed previously. Data are mean ± SEM.

Supplementary figure 5. Over-representation analysis of the differential gene expression profiles of PBMCs between the Fp100 and Ctr conditions (A), LPS and Ctr conditions (B) and Fp100 and LPS conditions (C) at 4 h on REACTOME pathway database. Interleukin_10_Signaling pathway is encircled in green, inflammatory pathways in pink and cell metabolism-related pathways in purple.

Supplementary figure 6. (A) Schematic representation of the two main energy metabolic pathways, glycolysis and mitochondrial respiration, including TCA cycle and oxidative phosphorylation. TCA, tricarboxylic acid cycle or Krebs cycle; ADP, adenine diphosphate; ATP, adenine triphosphate; NADH, Nicotinamide adenine dinucleotide; FADH2, *Flavin adenine dinucleotide.* (B) Schematic representation of the mitochondrial respiratory chain with the targets of each inhibitor used in the Seahorse Mito Stress assay. (C) Schematic representation of Oxygen Consumption Rate (OCR) evolution in time during a Mito Stress assay, showing basal respiration, ATP production, maximal respiration, spare capacity and non-mitochondrial respiration.

Suppl. Table 1. Characteristics of healthy individuals, controls and IBD patients recruited in the study. Continuous variables are presented as mean (SD). Categorical variables are presented as counts (%). Control: Healthy margin of patients operated on for colon adenocarcinoma. IBD treatment for ELISA experiments: 5-ASA, hydrocortisone, prednisone, tofacitinib; for flow cytometry experiments: 5-ASA, prednisone, azathioprine, adalimumab, tofacitinib and for RNAseq experiments: ustekinumab,

filgotinib. Abbreviation: IBD, inflammatory bowel disease; CD, Crohn disease; UC, ulcerative colitis; SD, standard deviation; NA, not applicable; BMI, body mass index.

	PBMC and CD14+ cells	ELISA on total mucosa		Flow cytometry on lamina propria		RNAseq on lamina propria $CD14^+$ cells
	Healthy subjects	IBD	Control	IBD	Control	IBD
n	28	8	8	3	4	3
UC/CD	NA	7/1	NA	2/1	NA	2/1
Sex [Male]	18 (64.3%)	4 (50%)	2 (25%)	2 (66.7%)	3 (75%)	2 (66.7%)
Age [years]	35.9 (11.2)	48.1 (16.1)	60.5 (21.2)	41.3 (11.2)	65.6 (34.6)	49.3 (26.8)
BMI [kg/m^2]	24.2 (5.3)	25 (7)	26.1 (8.2)	26.9 (12)	24.5 (3.4)	24.4 (1.9)
Patient with IBD treatment at time of surgery	NA	4 (50%)	NA	2 (66.7%)	NA	2 (66.7%)
Type of tissue analyzed						
Colon	NA	3	6	2	3	NA
Ileum	NA	7	2	2	1	3

Suppl. Table 1.

Suppl Fig 1

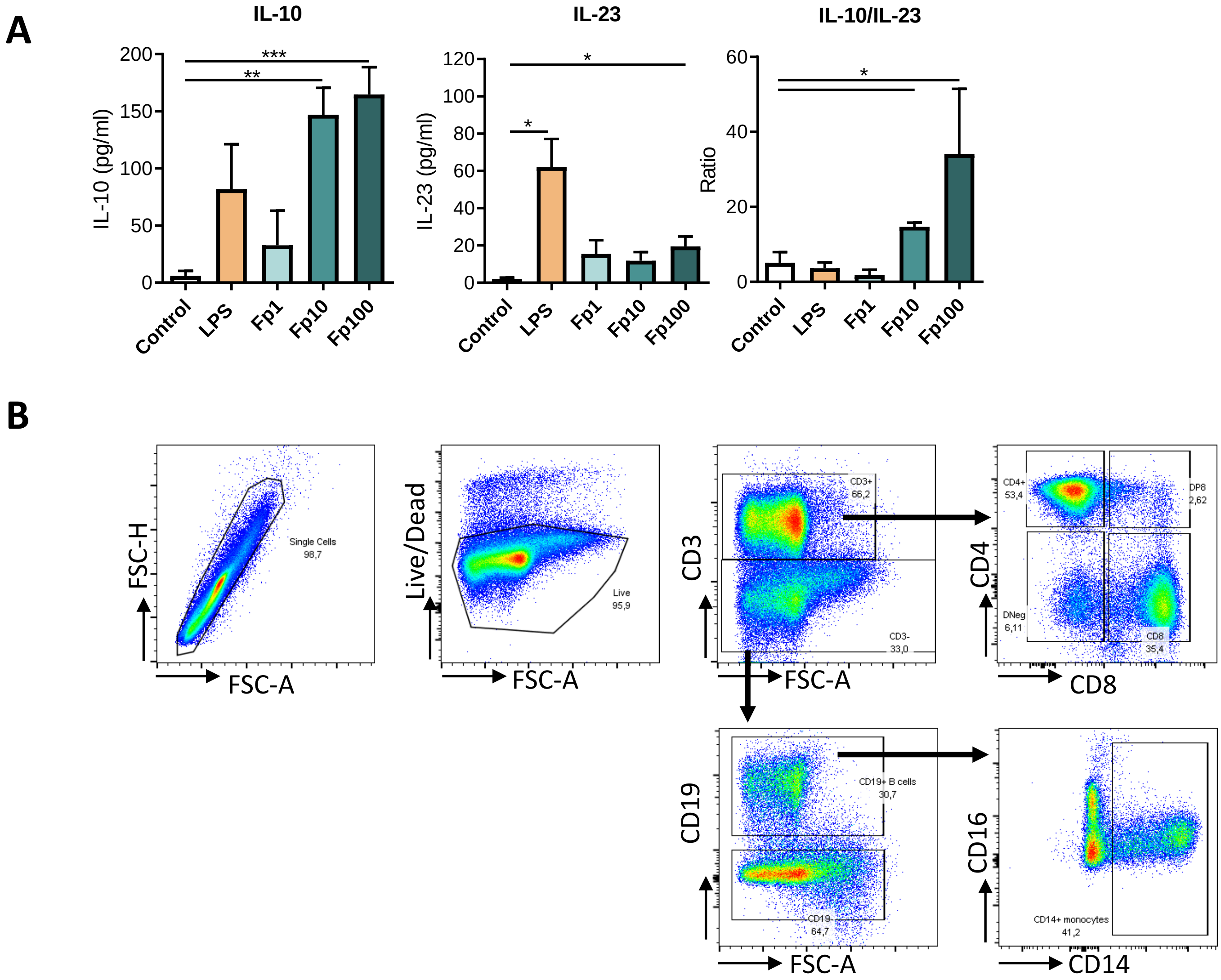
A
IL-10
IL-23
IL-10/IL-23
IL-10 (pg/ml)
IL-23 (pg/ml)
Ratio
Control
LPS
Fp1
Fp10
Fp100
B
FSC-H
FSC-A
Single Cells
98,7
Live/Dead
Live
95,9
CD3
CD3+
66,2
CD3-
33,0
CD4
CD8
CD4+
53,4
DP8
2,62
DNeg
6,11
CD8
35,4
CD19
CD19+ B cells
30,7
CD19-
64,7
CD16
CD14
CD14+ monocytes
41,2

Suppl Fig 2

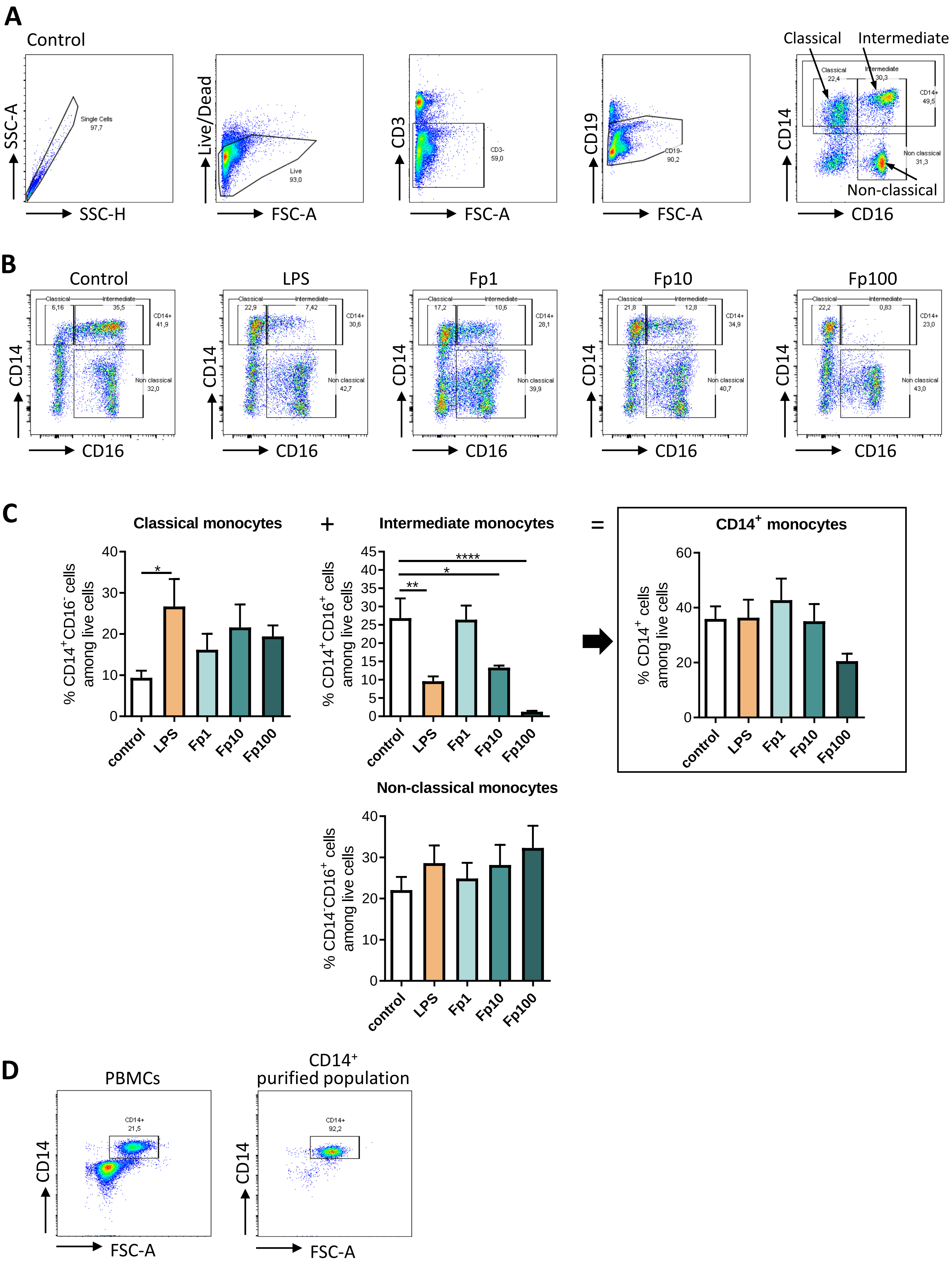
A
Control
Single Cells 97,7
SSC-A
SSC-H
Live/Dead
Live 93,0
FSC-A
CD3
CD3- 59,0
CD19
CD19- 90,2
Classical
Intermediate
Classical 22,4
Intermediate 30,3
CD14+ 49,5
Non classical 31,3
Non-classical
CD14
CD16
B
Control
LPS
Fp1
Fp10
Fp100
C
Classical monocytes
+
Intermediate monocytes
=
CD14+ monocytes
% CD14+CD16- cells among live cells
% CD14+CD16+ cells among live cells
% CD14+ cells among live cells
Non-classical monocytes
% CD14-CD16+ cells among live cells
control
LPS
Fp1
Fp10
Fp100
D
PBMCs
CD14+ purified population
CD14+ 21,5
CD14+ 92,2
CD14
FSC-A

Suppl Fig 3

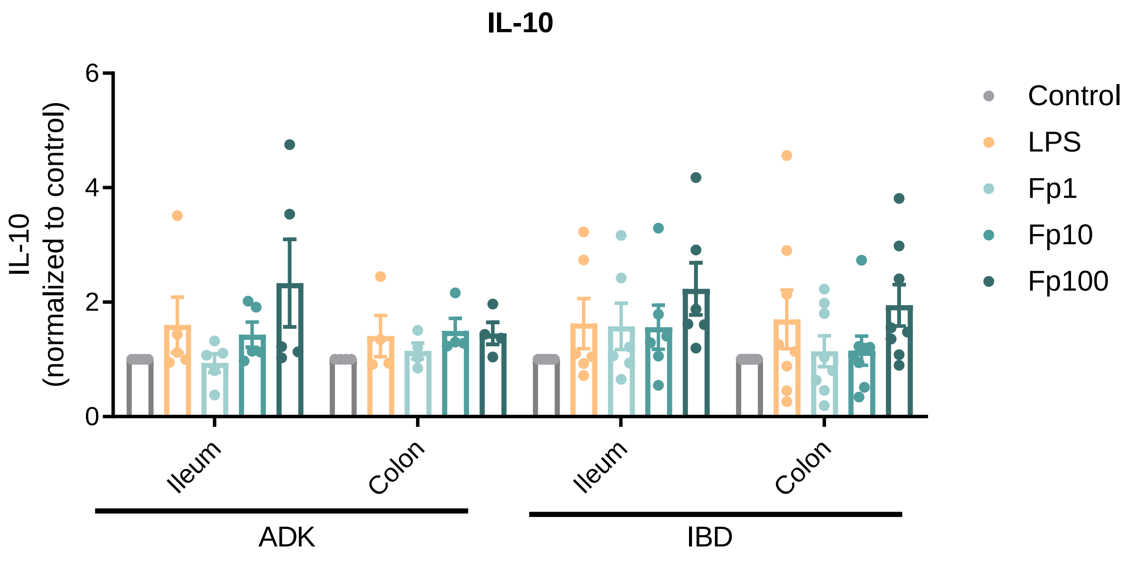

IL-10
IL-10
(normalized to control)
6
4
2
0
Ileum
Colon
Ileum
Colon
ADK
IBD
Control
LPS
Fp1
Fp10
Fp100

Suppl Fig 4

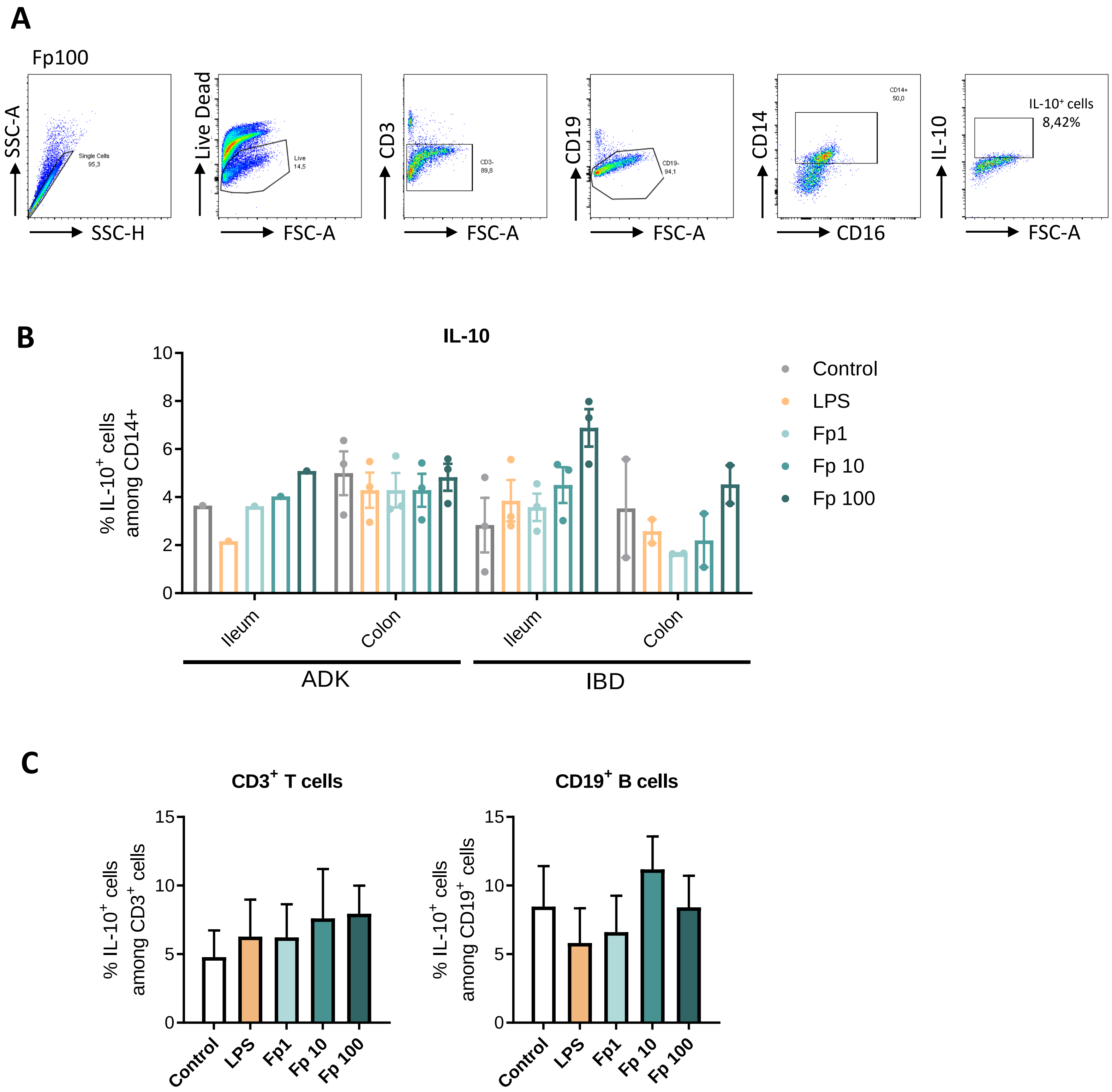

A
Fp100
Single Cells 95,3
SSC-A
SSC-H
Live Dead
Live 14,5
FSC-A
CD3
CD3- 89,8
FSC-A
CD19
CD19- 94,1
FSC-A
CD14
CD14+ 50,0
CD16
IL-10
IL-10⁺ cells 8,42%
FSC-A
B
IL-10
% IL-10⁺ cells among CD14+
10
8
6
4
2
0
Ileum
Colon
Ileum
Colon
ADK
IBD
Control
LPS
Fp1
Fp 10
Fp 100
C
CD3⁺ T cells
% IL-10⁺ cells among CD3⁺ cells
15
10
5
0
Control
LPS
Fp1
Fp 10
Fp 100
CD19⁺ B cells
% IL-10⁺ cells among CD19⁺ cells
15
10
5
0
Control
LPS
Fp1
Fp 10
Fp 100

Suppl Fig 5

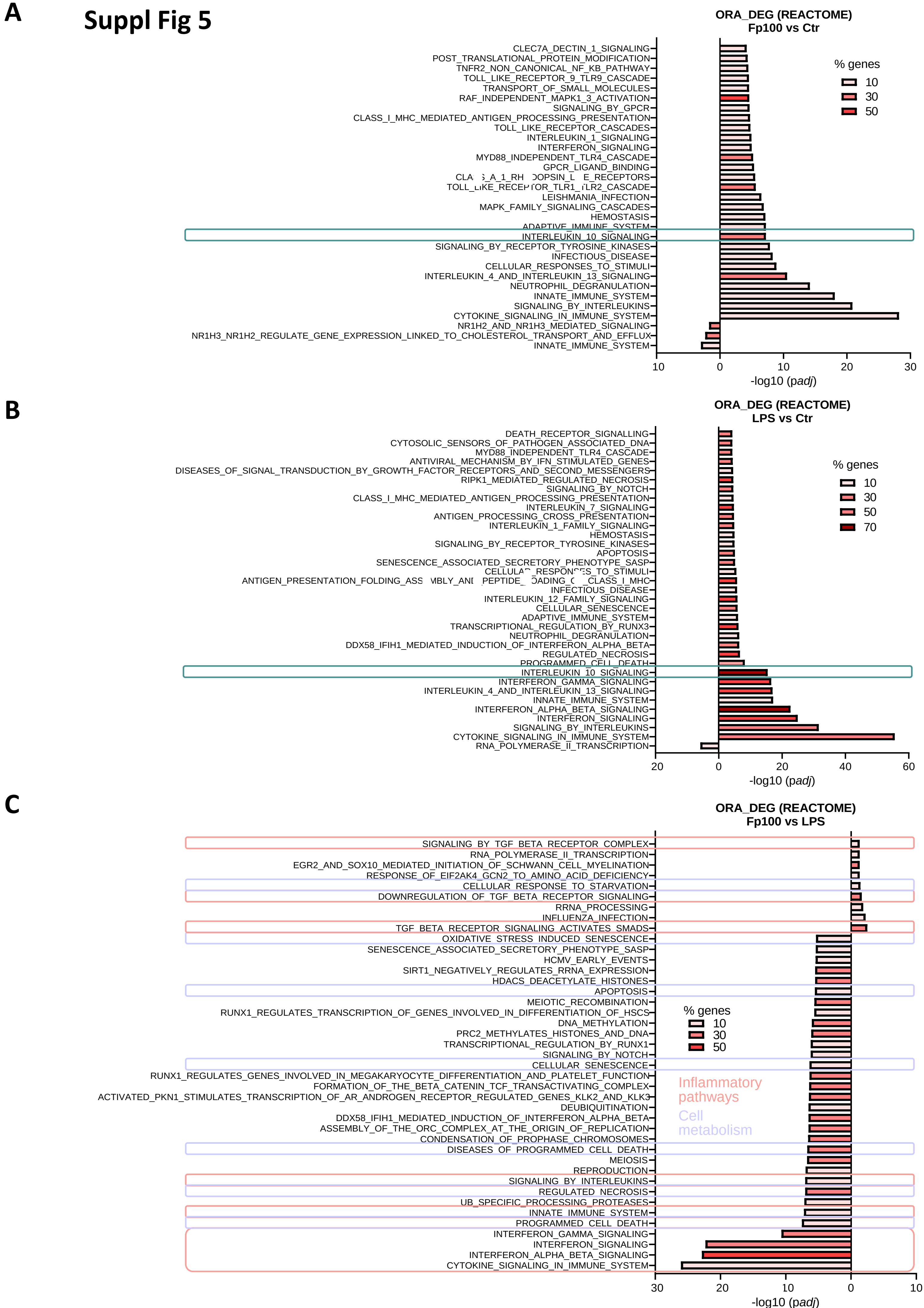
A
ORA_DEG (REACTOME)
Fp100 vs Ctr
CLEC7A_DECTIN_1_SIGNALING
POST_TRANSLATIONAL_PROTEIN_MODIFICATION
TNFR2_NON_CANONICAL_NF_KB_PATHWAY
TOLL_LIKE_RECEPTOR_9_TLR9_CASCADE
TRANSPORT_OF_SMALL_MOLECULES
RAF_INDEPENDENT_MAPK1_3_ACTIVATION
SIGNALING_BY_GPCR
CLASS_I_MHC_MEDIATED_ANTIGEN_PROCESSING_PRESENTATION
TOLL_LIKE_RECEPTOR_CASCADES
INTERLEUKIN_1_SIGNALING
INTERFERON_SIGNALING
MYD88_INDEPENDENT_TLR4_CASCADE
GPCR_LIGAND_BINDING
LEISHMANIA_INFECTION
MAPK_FAMILY_SIGNALING_CASCADES
HEMOSTASIS
ADAPTIVE_IMMUNE_SYSTEM
INTERLEUKIN_10_SIGNALING
SIGNALING_BY_RECEPTOR_TYROSINE_KINASES
INFECTIOUS_DISEASE
CELLULAR_RESPONSES_TO_STIMULI
INTERLEUKIN_4_AND_INTERLEUKIN_13_SIGNALING
NEUTROPHIL_DEGRANULATION
INNATE_IMMUNE_SYSTEM
SIGNALING_BY_INTERLEUKINS
CYTOKINE_SIGNALING_IN_IMMUNE_SYSTEM
NR1H2_AND_NR1H3_MEDIATED_SIGNALING
NR1H3_NR1H2_REGULATE_GENE_EXPRESSION_LINKED_TO_CHOLESTEROL_TRANSPORT_AND_EFFLUX
INNATE_IMMUNE_SYSTEM
% genes
10
30
50
10 0 10 20 30
-log10 (padj)
B
ORA_DEG (REACTOME)
LPS vs Ctr
DEATH_RECEPTOR_SIGNALLING
CYTOSOLIC_SENSORS_OF_PATHOGEN_ASSOCIATED_DNA
MYD88_INDEPENDENT_TLR4_CASCADE
ANTIVIRAL_MECHANISM_BY_IFN_STIMULATED_GENES
DISEASES_OF_SIGNAL_TRANSDUCTION_BY_GROWTH_FACTOR_RECEPTORS_AND_SECOND_MESSENGERS
RIPK1_MEDIATED_REGULATED_NECROSIS
SIGNALING_BY_NOTCH
CLASS_I_MHC_MEDIATED_ANTIGEN_PROCESSING_PRESENTATION
INTERLEUKIN_7_SIGNALING
ANTIGEN_PROCESSING_CROSS_PRESENTATION
INTERLEUKIN_1_FAMILY_SIGNALING
HEMOSTASIS
SIGNALING_BY_RECEPTOR_TYROSINE_KINASES
APOPTOSIS
SENESCENCE_ASSOCIATED_SECRETORY_PHENOTYPE_SASP
INFECTIOUS_DISEASE
INTERLEUKIN_12_FAMILY_SIGNALING
CELLULAR_SENESCENCE
ADAPTIVE_IMMUNE_SYSTEM
TRANSCRIPTIONAL_REGULATION_BY_RUNX3
NEUTROPHIL_DEGRANULATION
DDX58_IFIH1_MEDIATED_INDUCTION_OF_INTERFERON_ALPHA_BETA
REGULATED_NECROSIS
PROGRAMMED_CELL_DEATH
INTERLEUKIN_10_SIGNALING
INTERFERON_GAMMA_SIGNALING
INTERLEUKIN_4_AND_INTERLEUKIN_13_SIGNALING
INNATE_IMMUNE_SYSTEM
INTERFERON_ALPHA_BETA_SIGNALING
INTERFERON_SIGNALING
SIGNALING_BY_INTERLEUKINS
CYTOKINE_SIGNALING_IN_IMMUNE_SYSTEM
RNA_POLYMERASE_II_TRANSCRIPTION
% genes
10
30
50
70
20 0 20 40 60
-log10 (padj)
C
ORA_DEG (REACTOME)
Fp100 vs LPS
SIGNALING_BY_TGF_BETA_RECEPTOR_COMPLEX
RNA_POLYMERASE_II_TRANSCRIPTION
EGR2_AND_SOX10_MEDIATED_INITIATION_OF_SCHWANN_CELL_MYELINATION
RESPONSE_OF_EIF2AK4_GCN2_TO_AMINO_ACID_DEFICIENCY
CELLULAR_RESPONSE_TO_STARVATION
DOWNREGULATION_OF_TGF_BETA_RECEPTOR_SIGNALING
RRNA_PROCESSING
INFLUENZA_INFECTION
TGF_BETA_RECEPTOR_SIGNALING_ACTIVATES_SMADS
OXIDATIVE_STRESS_INDUCED_SENESCENCE
SENESCENCE_ASSOCIATED_SECRETORY_PHENOTYPE_SASP
HCMV_EARLY_EVENTS
SIRT1_NEGATIVELY_REGULATES_RRNA_EXPRESSION
HDACS_DEACETYLATE_HISTONES
APOPTOSIS
MEIOTIC_RECOMBINATION
RUNX1_REGULATES_TRANSCRIPTION_OF_GENES_INVOLVED_IN_DIFFERENTIATION_OF_HSCS
DNA_METHYLATION
PRC2_METHYLATES_HISTONES_AND_DNA
TRANSCRIPTIONAL_REGULATION_BY_RUNX1
SIGNALING_BY_NOTCH
CELLULAR_SENESCENCE
RUNX1_REGULATES_GENES_INVOLVED_IN_MEGAKARYOCYTE_DIFFERENTIATION_AND_PLATELET_FUNCTION
FORMATION_OF_THE_BETA_CATENIN_TCF_TRANSACTIVATING_COMPLEX
ACTIVATED_PKN1_STIMULATES_TRANSCRIPTION_OF_AR_ANDROGEN_RECEPTOR_REGULATED_GENES_KLK2_AND_KLK3
DEUBIQUITINATION
DDX58_IFIH1_MEDIATED_INDUCTION_OF_INTERFERON_ALPHA_BETA
ASSEMBLY_OF_THE_ORC_COMPLEX_AT_THE_ORIGIN_OF_REPLICATION
CONDENSATION_OF_PROPHASE_CHROMOSOMES
DISEASES_OF_PROGRAMMED_CELL_DEATH
MEIOSIS
REPRODUCTION
SIGNALING_BY_INTERLEUKINS
REGULATED_NECROSIS
UB_SPECIFIC_PROCESSING_PROTEASES
INNATE_IMMUNE_SYSTEM
PROGRAMMED_CELL_DEATH
INTERFERON_GAMMA_SIGNALING
INTERFERON_SIGNALING
INTERFERON_ALPHA_BETA_SIGNALING
CYTOKINE_SIGNALING_IN_IMMUNE_SYSTEM
% genes
10
30
50
Inflammatory pathways
Cell metabolism
30 20 10 0 10
-log10 (padj)

Suppl Fig 6

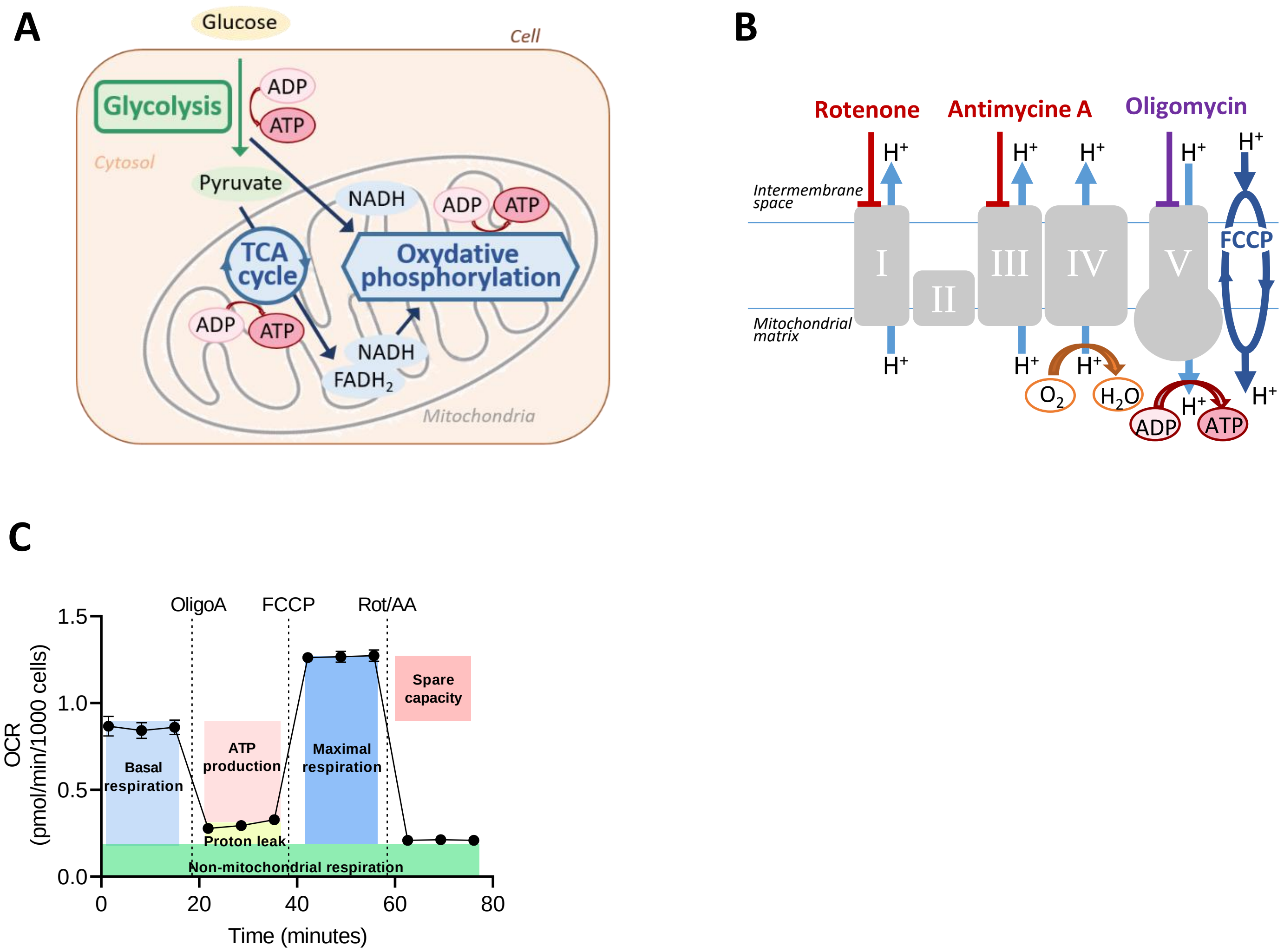
A
Glucose
Cell
Glycolysis
ADP
ATP
Cytosol
Pyruvate
NADH
ADP
ATP
TCA cycle
Oxydative phosphorylation
ADP
ATP
NADH
FADH2
Mitochondria
B
Rotenone
Antimycine A
Oligomycin
H+
Intermembrane space
I
II
III
IV
V
FCCP
Mitochondrial matrix
O2
H2O
ADP
ATP
C
OligoA
FCCP
Rot/AA
Spare capacity
ATP production
Maximal respiration
Basal respiration
Proton leak
Non-mitochondrial respiration
OCR (pmol/min/1000 cells)
1.5
1.0
0.5
0.0
0
20
40
60
80
Time (minutes)
