# Supplementary material for: Faecalibacterium prausnitzii Induces an Anti-inflammatory Response and a Metabolic Reprogramming in Human Monocytes
Source: Gastroenterology. 2026 Apr;170(4):704–20. doi: 10.1053/j.gastro.2025.10.003 (PMC13006548; doi:10.1053/j.gastro.2025.10.003)

Gastroenterology 2026;170:704–720

INFLAMMATORY BOWEL DISEASE

Faecalibacterium prausnitzii Induces an Anti-inflammatory Response and a Metabolic Reprogramming in Human Monocytes

Camille Danne,[1,2] Rodrigo de Oliveira Formiga,[1,2] Laura Creusot,[1,2] Florian Marquet,[1,2] Delphine Sedda,[3] Laura Hua,[3] Pauline Ruffié,[3] Hang-Phuong Pham,[4] Loic Brot,[1,2] Iria Alonso Salgueiro,[1,2] Marie-Laure Michel,[2,5] Philippe Langella,[2,5] Jérémie H. Lefevre,[2,6] **Harry Sokol,[1,2,5,§]** and **Nathalie Rolhion[1,2,§]**

[1]Centre de Recherche Saint-Antoine, CRSA, Sorbonne Université, Institut National de la Santé et de la Recherche Médicale, Gastroenterology Department, Assistance publique-Hôpitaux de Paris, Saint-Antoine Hospital, Paris, France; [2]Gut, Liver & Microbiome Research (GLIMMER) Fédération Hospitalo-Universitaire (FHU), Paris, France; [3]Exeliom Biosciences, Dijon, France; [4]Parean Biotechnologies, Saint-Malo, France; [5]Micalis Institute, Université Paris-Saclay, Institut National de Recherche pour l'Agriculture, l'Alimentation et l'Environnement (INRAE), AgroParisTech, Jouy-en-Josas, France; and [6]Department of Digestive Surgery, Assistance publique-Hôpitaux de Paris, Hôpital Saint Antoine, Sorbonne Université, Paris, France

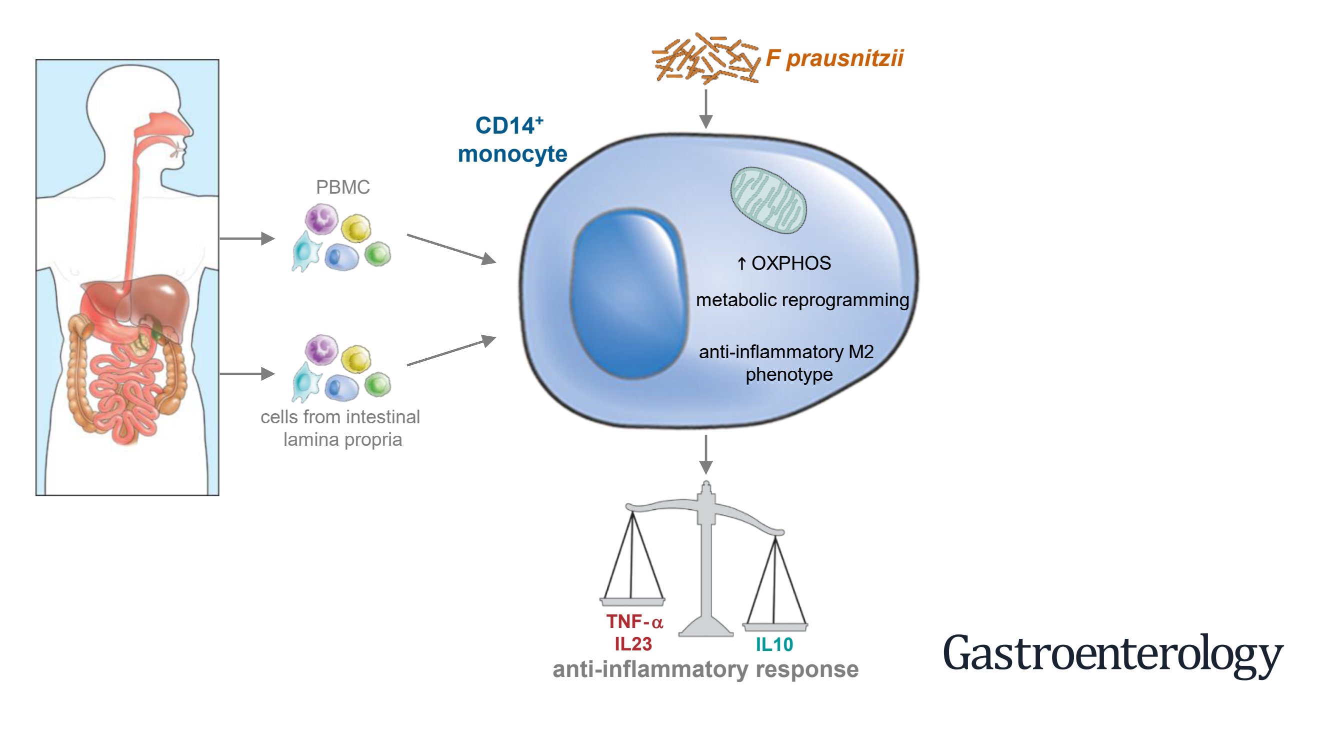

BACKGROUND & AIMS: *Faecalibacterium prausnitzii*, a highly abundant gut bacterium, has been linked to overall health, and is decreased in several pathologic conditions, such as inflammatory bowel disease (IBD). *F prausnitzii* has shown anti-inflammatory properties in human and mouse models, notably through the induction of interleukin 10 (IL10) signaling. Here, we investigated which cell types from human blood and intestinal tissue are responsible for producing IL10 induced by *F prausnitzii* and provide the first mechanistic insights. **METHODS:** Immune cells isolated from human blood and intestinal lamina propria of IBD patients and noninflamed controls, were stimulated with *F prausnitzii* EXL01 strain, *Coprococcus comes* 27758 strain, and *Escherichia coli* MG1655 strain, with or without lipopolysaccharide (LPS), and analyzed by LEGENDplex, enzyme-linked immunosorbent assay, flow cytometry, RNA sequencing, and Seahorse technology. **RESULTS:** *F prausnitzii* induced a direct and dose-dependent production of IL10 in cluster of differentiation 14$^+$ monocytes from the systemic circulation and intestinal tissue, without inducing a proinflammatory response compared with LPS stimulation. RNA sequencing and Seahorse analyses corroborated these results, revealing that *F prausnitzii* affects cellular energy metabolism in healthy and inflammatory conditions,

in a different way from 2 other tested bacteria and LPS. The anti-inflammatory response induced by *F prausnitzii* in monocytes was dependent on mitochondrial respiration. **CONCLUSIONS:** *F prausnitzii* induces an anti-inflammatory response and rewires energy metabolism in human monocytes in healthy and inflamed conditions, potentially explaining its beneficial impact on intestinal inflammation and human health in general. These results provide new insight into the mechanisms underlying the anti-inflammatory effects of *F prausnitzii* and are crucial for a better understanding of its potential use in IBD treatment.

Keywords: Faecalibacterium prausnitzii; Inflammatory Bowel Disease; IL10; CD14$^+$ Monocytes; Ileum.

Inflammatory bowel disease (IBD), including Crohn's disease and ulcerative colitis, are characterized by chronic and relapsing inflammation of the gastrointestinal tract, whose incidence and prevalence rates are increasing in both developed and developing countries.[1] There is a significant need to identify markers that could predict relapses and complications and develop new treatments, which would have considerable socioeconomic benefits. The exact pathogenesis of IBD remains enigmatic, notably due to its multifactorial etiology.

IBD partly results from genetic susceptibilities that are largely responsible for dysregulated immune responses toward commensal bacteria.[2–6] Additionally, environmental factors and lifestyle habits play a role, and the specific impact of gut microbiota alterations on IBD pathogenesis has been highlighted in numerous studies.[7,8] Indeed, the gut microbiota appears as both a marker of inflammation and an actor in IBD development. The proinflammatory effect of IBD-associated microbiota has been demonstrated by microbiota transfer from humans to mice[9] and by the positive effects of fecal microbiota transplantation used as a treatment of IBD in clinical trials.[10,11]

One of the most significant and consistent findings regarding the alterations of the gut microbiota in IBD is the decreased abundance of *Faecalibacterium prausnitzii*, a member of the Firmicutes phylum, belonging to the Clostridium IV group.[12–14] *F prausnitzii* is one of the most abundant bacterial species in the human intestinal microbiota of healthy adults.[15–19] Its high prevalence and abundance indicate its significant contribution to microbiota functions in healthy individuals. Indeed, high levels of *F prausnitzii* are associated with health, whereas low levels are linked to diseases such as IBD[18,19] and cancer.[20,21]

We recently showed that *F prausnitzii* EXL01 strain boosts efficacy of immune checkpoint inhibitors in vitro and in mouse models[20] and is currently being evaluated in several clinical trials in this indication (NCT06448572, NCT06551272, and NCT06253611). Although the molecular mechanisms involved are not fully understood, we demonstrated direct interactions between *F prausnitzii* EXL01 strain and host immune cells through activation of both dendritic cells and cluster of differentiation (CD) 4$^+$ T cells in the presence of immune checkpoint inhibitors. Moreover, *F prausnitzii* has shown immunomodulatory effects both in vitro and in vivo in different colitis mouse models.[18,22–24]

The anti-inflammatory effect of *F prausnitzii* notably relies on the induction of the production of the cytokine interleukin 10 (IL10) by immune cells, together with the low induction of proinflammatory cytokines.[18,25] *F prausnitzii* promotes tolerogenic IL10-producing dendritic cells that in turn favor the priming of IL10-producing CD4$^+$ T cells in vitro in human.[26] Moreover, *F prausnitzii* induces IL10-producing CD4$^+$CD8α^+ human regulatory T cells that

WHAT YOU NEED TO KNOW

BACKGROUND AND CONTEXT

Faecalibacterium prausnitzii has demonstrated anti-inflammatory properties in human cells and mouse models, notably by inducing interleukin 10 signaling. However, the specific immune cell types and underlying mechanisms involved remain unclear.

NEW FINDINGS

F prausnitzii directly induces interleukin 10 production by cluster of differentiation 14-positive human monocytes from the systemic circulation and intestinal tissue and reprograms the energy metabolism of these cells under healthy and inflammatory conditions. This differs from the effects observed with other tested bacteria and lipopolysaccharide.

LIMITATIONS

The bacterial molecules, host receptors, and signaling pathways involved remain to be explored.

CLINICAL RESEARCH RELEVANCE

F prausnitzii EXL01 strain is already in the clinical development stage for inflammatory bowel disease and is an ideal candidate for the development of live biotherapeutic products in the context of intestinal inflammation.

BASIC RESEARCH RELEVANCE

This study provides a better understanding of the anti-inflammatory properties of the *F prausnitzii* EXL01 strain by exploring the types of responding immune cells and providing the first mechanistic insights involved.

INFLAMMATORY BOWEL DISEASE

[§] Authors share co-senior authorship.

Abbreviations used in this paper: ADK, adenocarcinoma; ATP, adenosine 5′-triphosphate; CD, cluster of differentiation; DP8, CD4$^+$CD8$^+$ double-positive T cells; ELISA, enzyme-linked immunosorbent assay; FCCP, carbonylcyanide *p*-trifluoromethoxyphenylhydrazone; Fp, *Faecalibacterium prausnitzii*; IBD, inflammatory bowel disease; IL10, interleukin 10; LPS, lipopolysaccharide; MOI, multiplicity of infection; ORA, over-representation analysis; OXPHOS, oxidative phosphorylation; PBMC, peripheral blood mononuclear cell; PBS, phosphate-buffered saline; RNAseq, RNA sequencing; TCA, tricarboxylic acid; TNF-α, tumor necrosis factor-α.

Most current article

© 2026 The Author(s). Published by Elsevier Inc. on behalf of the AGA Institute. This is an open access article under the CC BY license (http://creativecommons.org/licenses/by/4.0/).
0016-5085
https://doi.org/10.1053/j.gastro.2025.10.003

protect against intestinal inflammation in a humanized murine model.[19,27,28] However, the complete picture of the cell types responding to *F prausnitzii* by producing IL10 in humans, and the mechanisms at play, remain unknown.

This work used *F prausnitzii* EXL01 strain, already in clinical development for IBD (NCT05542355 and NCT06925061). We found that CD14$^+$ monocytes, isolated from both human blood and intestinal tissue from IBD patients and noninflamed controls, are the main producers of IL10 in response to *F prausnitzii* EXL01 strain, with a lower production of IL23 and tumor necrosis factor-α (TNF-α) compared with lipopolysaccharide (LPS). Using RNA sequencing (RNAseq), we found that *F prausnitzii* EXL01 strain does not induce the proinflammatory response triggered by the classical bacterial factor LPS. In addition, the *F prausnitzii* EXL01 strain acts on the cell energy metabolism of human CD14$^+$ monocytes, inducing oxidative phosphorylation (OXPHOS) and inhibiting glycolysis and several cell death-related pathways compared with LPS stimulation.

Using Seahorse technology, we confirmed that the anti-inflammatory response to *F prausnitzii* EXL01 strain relies on mitochondria and on the rewiring of cellular energy metabolism in human CD14$^+$ monocytes. These effects were not observed when cells were stimulated with other bacteria, suggesting a specific effect of *F prausnitzii* EXL01 strain. We also showed that *F prausnitzii* affects cellular energy metabolism in healthy and inflammatory conditions in a different way from other tested bacteria and LPS. This study provides new insights into the mechanism underlying the anti-inflammatory effects of *F prausnitzii* on human immune cells.

Materials and Methods

Human Samples

All human samples were collected with informed consent. Approval for human studies was obtained from the local ethics committee (Comité de Protection de Personnes Ile de France IV, IRB 00003835 Suivitheque study; registration number 2012/05NICB + Comité de Protection de Personnes Ile de France III, Biomhost study; EUDRACT number 2018-A02978-47-18.1). Blood from healthy volunteers was obtained from a blood bank (Etablissement Français du Sang, convention C CPSL UNT—No.15/EFS/020) or from the Suivithèque study. All patients (both IBD and colon adenocarcinoma [ADK] patients) from the BiomHost study were recruited in the Department of Digestive Surgery of the Saint Antoine Hospital (Paris, France) between October 2022 and October 2023. Characteristics of healthy individuals and patients recruited to the study are presented in Supplementary Table 1.

Bacterial Culture

The *F prausnitzii* EXL01 strain was grown at 37°C in an Exeliom Bisociences' proprietary medium according to an in-house production process by Exeliom Biosciences. The test material was prepared to be ready-to-use in phosphate-buffered saline (PBS), characterized by enumerating the number of morphologically intact bacteria (ie, the total cell count).

Coprococcus comes (ATCC 27758) was grown 48 hours at 37°C in anaerobic conditions (90% N_2, 5% CO_2, and 5% H_2) in brain-heart infusion medium (Millipore) supplemented with yeast extract (5 g/L, Millipore), cellobiose (1 g/L; Sigma-Aldrich), maltose (1 g/L, Sigma-Aldrich), L-cysteine (0.5 g/L, Sigma-Aldrich), hemin (10 μmol/L, Sigma-Aldrich), vitamin K_1 (2.2 μmol/L Sigma-Aldrich), and vitamin K_3 (18.6 μmol/L, Sigma-Aldrich). *C comes* was enumerated using BactoBox (SBT Instruments), pelleted, weighted, resuspended in PBS, and stored at –80°C. EXL01 and *C comes* were exposed to oxygen during samples preparation and were therefore metabolically inactivated.

Escherichia coli strain MG1655 was grown overnight at 37°C in LB broth (Sigma-Aldrich), enumerated using BactoBox, pelleted, weighted, resuspended in PBS, heat-killed 30 minutes at 80°C, and stored at –80°C. The unculturability of the 3 bacteria was checked on agar plates.

Isolation of Peripheral Blood Mononuclear Cells and Cluster of Differentiation 14-Positive Cells

Fresh peripheral blood mononuclear cells (PBMCs) were isolated from human blood using Histopaque-1077 (Sigma-Aldrich) and Sepmate-50 tubes (STEMCELL Technologies) according to the manufacturer's recommendations. PBMCs were directly stimulated or counted to isolate CD14$^+$ monocytes using human CD14 MicroBeads and mass spectrometry columns on a MiniMACS Separator (Miltenyi Biotech).

Human Lamina Propria Cells Isolation From Intestinal Resection

The intestinal mucosa was dissociated from the resection and washed for 15 minutes in a mixture of antibiotics and antifungal agents (100 U/mL penicillin/streptomycin, 100 μg/mL gentamycin, and 0.1 μg/mL amphotericin B). Fragments of 0.5 mm^2 were cut and incubated 2 consecutive times in dissociation medium (PBS 1X + HEPES 10 mmol/L + EDTA 5 mmol/L + Roswell Park Memorial Institute 2%) for 15 minutes at 37°C under 100 rpm agitation. Tissue was harvested and incubated in digestion buffer (Roswell Park Memorial Institute + fetal calf serum 2% + 0.5 mg/mL deoxyribonuclease I [Sigma 11284932001] + collagenase IV [0.5 mg/mL, Sigma C5138]) for 40 minutes at 37°C under 100 rpm agitation. The supernatant was filtered, and lamina propria immune cells were counted for direct stimulation or isolation of CD14$^+$ cells, as described in the section above.

Cells Stimulation

Total human PBMCs (5×10^5), intestinal lamina propria (5×10^5), or CD14$^+$ isolated cells (1×10^5) were seeded in RPMI 1640 Medium Glutamax (Gibco, Thermo Fisher Scientific) supplemented with 10% heat-inactivated fetal bovine serum, 1% penicillin/streptomycin (10,000 U/mL, Gibco), 1 mmol/L pyruvate (Gibco) and 0.05 μmol/L β-mercapthoethanol (Sigma-Aldrich). Cells were then stimulated with control medium, *E coli* K12 LPS (100 ng/mL) or EXL01 strain at 3 doses (multiplicity of infection [MOI] 1, 10, and 100) for 6 or 16

hours at 37°C in a 5% CO_2 incubator, as specified in the figure legends.

LEGENDplex and Enzyme-Linked Immunosorbent Assay

LEGENDplex (Human Inflammation Panel 1, BioLegend) and enzyme-linked immunosorbent assay (ELISA; Invitrogen, Thermo Fisher Scientific) assays on cell supernatants were performed according to the manufacturers' recommendations. Human Inflammation Panel 1 enables the simultaneous quantification of 13 human inflammatory cytokines: IL1β, interferon-α2, interferon-γ, TNF-α, C-C motif chemokine ligand 2, IL6, chemokine (C-X-C motif) ligand 8, IL10, IL12p70, IL17A, IL18, IL23, and IL33. LEGENDplex experiments were acquired on a BD Accuri C6 flow cytometer and analyzed using the online software Qognit. To normalized ELISA assays, Cytotoxicity Detection Kit (lactate dehydrogenase, 11644793001, Roche) was used at the end of each experiment on 25 μL cell supernatant.

Flow Cytometry

Extracellular staining was performed in fluorescence-activated cell sorter buffer (PBS1X + EDTA 0.5 mmol/L + fetal calf serum 2%). Staining includes a viability dye (Zombie Aqua Fixable Viability Kit, BioLegend) and the following antibodies: CD4 (BV605, SK3, BioLegend 344646), CD8 (BV510, SK1, BioLegend 344732), CD3 (PB, OKT3, BioLegend 317314), CD19 (AF700, SJ25C1, BioLegend 363024), CD14 (PE/Dazzle 594, HCD14, BioLegend 325634), CD16 (BV785, 3G8, BioLegend 302046), HLA-DR (APC, L243, BioLegend 307610), and Live/Dead (Zombie NIR Fixable Viability Kit, BioLegend 423105) for the PBMCs panel, and CD14 (PE/Dazzle 594, HCD14, BioLegend 325634), CD3 (APCvio770, RE1613, Miltenyi Biotec 130113136), CD19 (BV605, HIB19, BioLegend 302244), and CD16 (BV785, 3G8, BioLegend 302046) for the $CD14^+$ monocytes panels. For intracellular staining, Cytofix/Cytoperm (BD) was used following the manufacturer's protocols with the following antibodies: IL10 (phycoerythrin, FES3-9D7, BioLegend 501404), IL23 (DyLight488, Novus 727753, FAB1716k), and TNF-α (AF700, MAb11, BioLegend 502928). All data were acquired with a CytoFlex (BD) and analyzed with FlowJo software.

RNA Sequencing Analysis

RNA was extracted from 1×10^5 $CD14^+$ monocytes from 6 healthy individuals using the QIAGEN RNeasy kit, and RNAseq (including a Smarter stranded V3 library preparation step) was performed at the platform Genotyping/Sequencing, Institut du Cerveau et de la Moelle Epinière (ICM) Paris Brain Institute (Hôpital de la Pitié-Salpêtrière CNRS UMR 7225—INSERM U 1127—Sorbonne Université UM75) on an Illumina NovaSeq X sequencer. Illumina's adapters and bad quality bases (Phred <20) were removed from reads using TrimGalore 0.6.7.[29] fastQC 0.12.0 was used for the quality control of raw and trimmed paired-end fastqs. Salmon 1.10.1[30] was used to quantify reads against a mapping-based index built from the GENCODE 45 (Genome Reference Consortium Human Build 38) transcript set.[31]

Quantified transcripts were imported into R software (R Foundation for Statistical Computing) using the package tximport 1.30.0.[32] Gene-level *DESeqDataSet* object was built from previously imported transcript abundances using the package DESeq2 1.42.1[33] to perform the differential expression analysis. A prefiltering was applied to remove (1) genes having 0 count in >60% of samples within one of the biological conditions and (2) genes without an official symbol.

For visualization, raw counts were transformed by the "variance stabilizing transformation" method. Principal component analysis was performed on variance stabilizing transformation and normalized count dataset using the function *prcomp* to explore the relationship between biological conditions. Differential expression analysis was performed by the function *DESeq* of the package DESeq2. Briefly, (1) the size factor was estimated for each sample, (2) the dispersion was estimated for each gene, (3) all counts were fitted to a negative binomial generalized linear model, and (4) contrasts and Wald's test were performed for testing the difference of expression between biological conditions.

Venn's diagrams were used to visualize the overlap between significant genes lists through the package ggvenn 0.1.10. In case of 3 samples by group, EBSeq 2.0.0[34] was used for differential analysis as advised by Li et al.[35] Biological function and pathways enriched by significant genes were assessed using over-representation analysis (ORA) on Hallmark and Reactome database through the package msigdbr 7.5.1. ORA was performed separately for significantly up-regulated genes (logfold change >0) and down-regulated genes (logfold change <0) in each comparison using the function *fora* from the R package fgsea.[36]

In addition, pathways were tested for their significant enrichment between biological conditions by gene set enrichment analysis[37] using the function *fgsea* from the R package fgsea. Plots were produced by the package ggplot2 3.5.1. The Benjamini-Hochberg method was used for controlling the false-discovery rate. Significant level was fixed at type I error $\alpha = 0.05$.

Seahorse Experiments

Mito stress test assay was performed on a XF96 Extracellular Flux Analyzer (Seahorse Biosciences). $CD14^+$ monocytes (1×10^5 cells/well) were isolated from fresh human PBMCs from 3 healthy donors and stimulated for 6 hours with control medium, LPS (100 ng/mL), or EXL01 strain at MOI 100 or with the same amount of *C comes* or *E coli* on a dry weight basis. Supernatants were collected for ELISA and lactate dehydrogenase assays, as described above.

Cells were washed and seeded in poly-D-lysine precoated Seahorse plates with Agilent Seahorse XF RPMI Medium, pH 7.4, supplemented with XF 10 mmol/L glucose solution, XF 1 mmol/L pyruvate solution, and XF 2 mmol/L glutamine. Then, cells were washed in Seahorse RPMI medium and incubated for 1 h at 37°C without CO_2. In the analyzer, oligomycin 1.5 μmol/L, carbonylcyanide *p*-trifluoromethoxyphenylhydrazone (FCCP) 1 μmol/L, and rotenone + antimycin A 0.5 μmol/L were injected at the indicated times. Groups were set as quadruplicates, and standardization was performed after each experiment using Hoescht labeling of cells and counting in a Cytation 5

microscope (Agilent BioTek), with no noticeable differences in cell numbers according to the stimulation conditions.

Statistics

Data were analyzed using GraphPad Prism 8 (GraphPad Software, San Diego, CA). Values are expressed as mean ± standard error of the mean. When only 1 factor was considered in the statistical analysis, a 1-way analysis of variance (ANOVA) was used. When 2 factors (ileum or colon topography and bacterial dose) were considered, a 2-way ANOVA was used. When some data were missing, a mixed-effects analysis was used instead of a repeated-measures ANOVA. Multiple comparisons were corrected using Dunnett's test. If not specified, the analysis was performed only against the control conditions. The statistical tests used are indicated in the figure legends.

Results

Faecalibacterium prausnitzii *EXL01 Strain Induces the Production of Interleukin 10 by Human Cluster of Differentiation 14-Positive Monocytes*

To investigate the immunomodulatory properties of *F prausnitzii* in humans, PBMCs were isolated from the fresh blood of 6 healthy volunteers and stimulated for 16 hours with *F prausnitzii* EXL01 strain at different MOIs (1, 10 and 100; corresponding to Fp1, Fp10, Fp100 in Figures) or with *E coli* K12 LPS (100 ng/mL), used as a positive control (Figure 1). Release of cytokines in cell supernatant was evaluated using LEGENDplex, an unbiased approach allowing the simultaneous quantification of several human inflammatory cytokines (both pro- and anti-inflammatory). The *F prausnitzii* EXL01 strain induced the secretion of the anti-inflammatory cytokine IL10 in a dose-dependent manner, whereas the induction of the proinflammatory cytokines such as IL23 or TNF-α was reduced compared with the LPS condition.

To provide a more clinically relevant picture, cytokine ratios were calculated and the *F prausnitzii* EXL01 strain induced an increased IL10/IL23 and IL10/TNF-α anti-inflammatory ratios (Figure 1*A*). ELISA analyses performed independently confirmed this result (Supplementary Figure 1*A*). Of note, LEGENDPlex analysis revealed a dose-dependent increase in the secretion of the proinflammatory cytokine IL12p70 when PBMCs were stimulated by *F prausnitzii* (Figure 1*A*). However, the absolute level of this cytokine was low (in the 10s of pg/mL range), and ELISA did not confirm this increase because the cytokine concentrations were below the detection limit. Therefore, further investigations into IL12p70 were not pursued; instead, the focus was shifted to cytokines with higher secretion levels and greater physiological relevance.

To identify the specific cell types involved in this immune response, stimulated PBMCs were analyzed by flow cytometry after IL10 intracellular staining (Supplementary Figure 1*B*). The *F prausnitzii* EXL01 strain induced a dose-dependent increase of the percentage of IL10$^+$ cells in CD14$^+$ monocytes (reaching ~20% of IL10$^+$ cells among CD14$^+$ cells stimulated with MOI 100). A significant increase was also observed in CD4$^+$CD8$^+$ double-positive T cells (DP8) but to a lesser extent (reaching ~5% of IL10$^+$ cells among DP8 stimulated with MOI100). An increase was observed in CD8$^+$ T cells but was not significant (reaching ~4% of IL10$^+$ cells among CD8$^+$ T cells stimulated with MOI 100) (Figure 1*B*).

Faecalibacterium prausnitzii *EXL01 Strain Directly Induces the Production of Interleukin 10 in Cluster of Differentiation 14-Positive Monocytes From Systemic Circulation*

Intracellular staining was performed for the 3 cytokines IL10, IL23, and TNF-α together with an antibody panel differentiating classical (CD14$^+$CD16$^-$), intermediate (CD14$^+$CD16$^+$), and nonclassical (CD14$^-$CD16$^+$) monocyte populations (Supplementary Figure 2*A*). As observed with LPS, the stimulation of PBMCs with *F prausnitzii* EXL01 strain increased the percentage of classical and nonclassical monocytes and significantly reduced the percentage of intermediate monocytes, leading to a slight decrease in the percentage of total CD14$^+$ monocytes (including classical and intermediate populations) (Supplementary Figure 2*B* and *C*). *F prausnitzii* EXL01 strain induced an increase in IL10$^+$CD14$^+$ monocytes, and a slight increase of IL23$^+$CD14$^+$ monocytes, but no increase in TNF-α^+CD14$^+$ monocytes, leading to a dose-dependent but not statistically significant increase in both IL10/IL23 and IL10/TNF-α anti-inflammatory ratios (Figure 2*A*). Similar trends are obtained for the 3 different monocytes subtypes taken individually (data not shown).

Moreover, the stimulation of CD14$^+$ monocytes purified from total human PBMCs recapitulated the capacity of *F prausnitzii* EXL01 strain to induce IL10 secretion by these cells, together with low production of IL23 and TNF-α compared with LPS, leading to a dose-dependent increase in anti-inflammatory ratios (Figure 2*B* and Supplementary Figure 2*D*). Interestingly, the lower dose of *F prausnitzii* EXL01 strain Fp1 was sufficient to induce maximal production of IL10 in CD14$^+$ monocytes, and increasing doses reduced the induction of proinflammatory cytokines in a dose-dependent manner (Figure 2*B*). Thus, the production of IL10 in CD14$^+$ monocytes relies on a direct stimulation by *F prausnitzii* (bacterium or its components) and does not depend on other immune cell types (Figure 2*B*).

Faecalibacterium prausnitzii *Induces the Production of Interleukin 10 in Cluster of Differentiation 14-Positive Monocytes From Intestinal Tissue of Patients With Inflammatory Bowel Disease and Noninflamed Controls*

To evaluate the immunomodulatory properties of *F prausnitzii* EXL01 strain directly in a more relevant clinical setup, including in pathological conditions, we took advantage of intestinal tissues, both ileum and colon, from

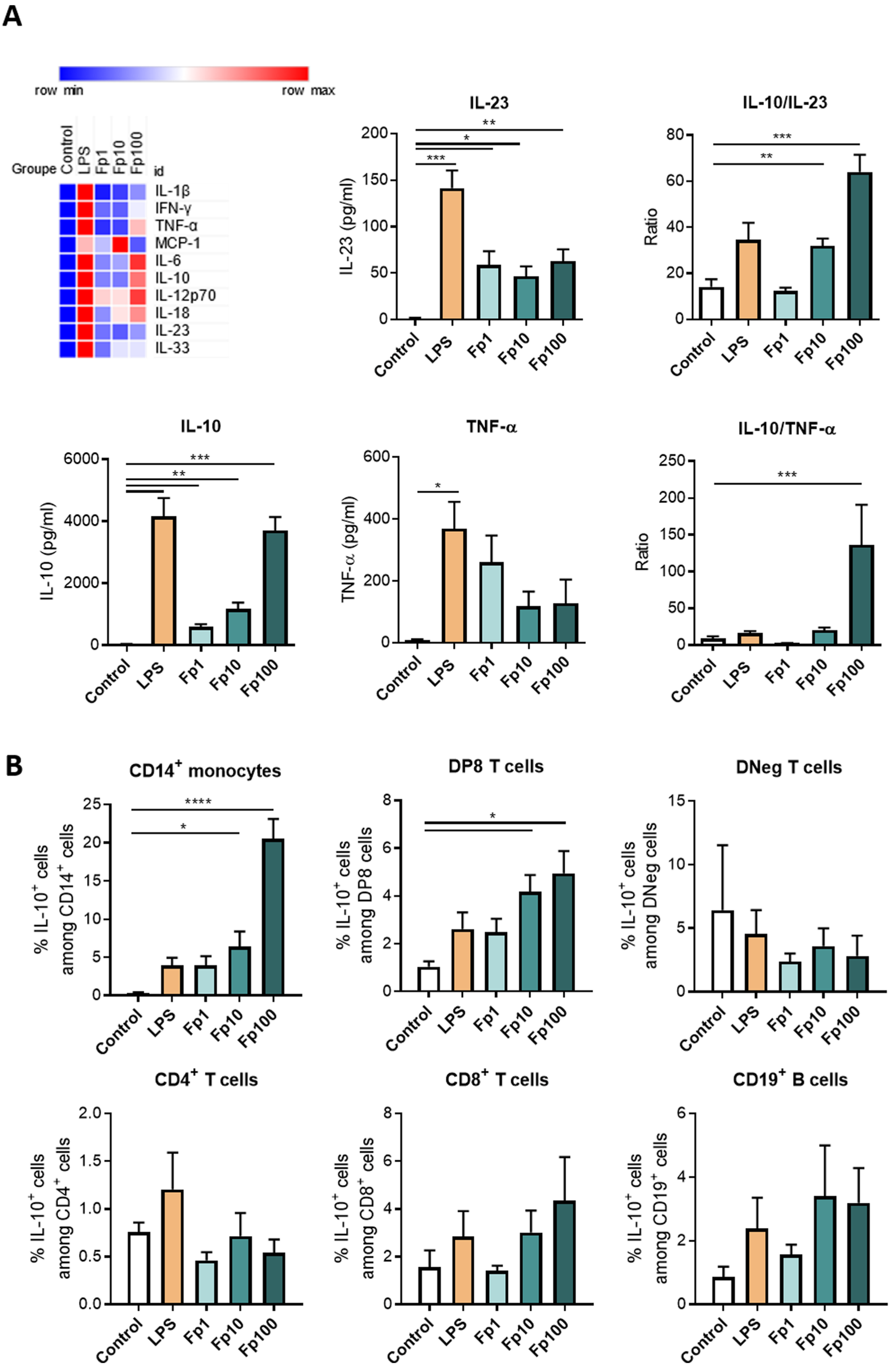
A
row min
row max
Groupe
Control
LPS
Fp1
Fp10
Fp100
id
IL-1β
IFN-γ
TNF-α
MCP-1
IL-6
IL-10
IL-12p70
IL-18
IL-23
IL-33
IL-23
IL-23 (pg/ml)
IL-10/IL-23
Ratio
IL-10
IL-10 (pg/ml)
TNF-α
TNF-α (pg/ml)
IL-10/TNF-α
Ratio
B
CD14+ monocytes
% IL-10+ cells among CD14+ cells
DP8 T cells
% IL-10+ cells among DP8 cells
DNeg T cells
% IL-10+ cells among DNeg cells
CD4+ T cells
% IL-10+ cells among CD4+ cells
CD8+ T cells
% IL-10+ cells among CD8+ cells
CD19+ B cells
% IL-10+ cells among CD19+ cells
Control
LPS
Fp1
Fp10
Fp100

patients operated on for IBD or colon ADK. We used inflamed but nonulcerated intestinal tissue from patients with IBD, and tumor-free healthy margins of ADK patients as noninflamed controls. Intestinal mucosa containing epithelial and lamina propria immune cells were isolated and 0.5 mm^2 pieces were stimulated with 3 doses of *F prausnitzii* EXL01 strain (as used in our cellular in vitro assay: Fp1, Fp10, Fp100) or with LPS for 16 hours (Figure 3). Despite a complex tissue structure and a large cellular diversity, *F prausnitzii* EXL01 strain induced IL10 secretion both in total ileum and colon mucosa (Figure 3*B*), and similarly in IBD and ADK patients (Supplementary Figure 3). In comparison, the proinflammatory cytokines IL23 and TNF-α were only slightly induced in response to the *F prausnitzii* EXL01 strain in this model, leading to dose-dependent increases in anti-inflammatory ratios (significant only for IL10/IL23 in ileum) (Figure 3*C*).

To directly assess the response of intestinal immune cells to *F prausnitzii* EXL01 strain, immune cells were isolated from fresh surgical tissue lamina propria and were stimulated in similar conditions (Figure 4). *F prausnitzii* EXL01 strain induced an increase in $IL10^+CD14^+$ monocytes in a dose-dependent manner mostly in ileum cells and similarly in IBD and ADK patients (Figure 4*B* and Supplementary Figure 4*A* and *B*). *F prausnitzii* EXL01 strain induced a decrease in $IL23^+CD14^+$ or TNF-α^+CD14^+ monocytes especially in ileum, leading to increased anti-inflammatory ratios in ileum (Figure 4*C* and *D*). As observed with PBMCs (Figure 1*B*), *F prausnitzii* EXL01 strain did not induce the production of IL10 in $CD3^+$ T cells and $CD19^+$ B cells isolated from intestinal tissue in our assay (Supplementary Figure 4*C*).

Altogether, these results confirm that *F prausnitzii* EXL01 strain triggers an anti-inflammatory response in $CD14^+$ monocytes from both the systemic circulation and the human intestinal tissue.

Faecalibacterium prausnitzii *EXL01 Strain Differentially Affects Immune Response and Cell Energy Metabolism Compared With Lipopolysaccharide*

To get an insight into the mechanisms underlying the anti-inflammatory effects of *F prausnitzii* on $CD14^+$ monocytes, RNAseq analysis was performed on $CD14^+$ cells sorted from human PBMCs (Figure 5 and Supplementary Figure 5) and stimulated with *F prausnitzii* EXL01 strain (Fp10 and Fp100) or LPS. Principal component analysis showed that stimulation of $CD14^+$ monocytes with *F prausnitzii* exl01 strain induced a different gene expression profile compared with the classical bacterial factor LPS (Figure 5*B*). Functional analysis (ORA of differentially expressed gene, Reactome pathway database) confirmed that both *F prausnitzii* EXL01 strain and LPS could induce the IL10 signaling pathway (Supplementary Figure 5*A* and *B*). However, many pro-inflammatory pathways, such as Interferon_Gamma_Response, Interferon_Alpha_Response, Tnfa_Signaling_Via_Nfkb, Inflammatory_Response, Complement, Il6_Jak_Stat3_Signaling and IL2_Stat5_Signaling, were significantly downregulated by *F prausnitzii* EXL01 strain compared with LPS (ORA, differentially expressed gene Reactome and Hallmark pathway databases) (Figure 5*C* and Supplementary Figure 5*C*). Interestingly, cell metabolism-related pathways were also differentially regulated between the 2 conditions, with an up-regulation of Oxydative_Phosphorylation and a down-regulation of Apoptosis in the Fp100 condition vs LPS (Figure 5*C*). Difference-in-differences analysis supported the finding that the *F prausnitzii* EXL01 strain did not induce pro-inflammatory pathways compared with LPS, but up-regulated Oxidative Phosphorylation while down-regulating Glycolysis and Apoptosis pathways (Figure 5*D* and *E*).

To go further, RNAseq analysis on $CD14^+$ monocytes isolated from ileum lamina propria of patients with IBD (N = 3) was performed in similar experimental conditions (Figure 5*F*). The activation of the IL10 signaling pathway by *F prausnitzii* EXL01 strain was confirmed (gene set enrichment analysis, Reactome database) (Figure 5*G*). Moreover, difference-in-differences analysis pointed out a down-regulation of pro-inflammatory pathways, including Interferon_Gamma_Response, Interferon_Alpha_Response, Tnfa_Signaling_Via_Nfkb, Inflammatory_Response and Il6_Jak_Stat3_Signaling in Fp100 compared with LPS, together with a down-regulation of Apoptosis (Figure 5*H*).

Anti-inflammatory Effects of Faecalibacterium prausnitzii *EXL01 Strain Relies on Rewiring Energy Metabolism in Cluster of Differentiation 14-Positive Monocytes*

Guided by RNAseq results, we further explored the impact of *F prausnitzii* EXL01 strain on the energy metabolism of $CD14^+$ monocytes. Cells isolated from fresh human PBMCs were stimulated in the presence or absence of oligomycin, a complex V mitochondrial respiration inhibitor (Figure 6*A* and Supplementary Figure 6*A*–*C*).

Figure 1. *Faecalibacterium prausnitzii* EXL01 strain induces the production of IL10 in PBMCs from systemic circulation. (*A*) PBMCs from the fresh blood of healthy volunteers were isolated and stimulated for 16 hours with *F prausnitzii* EXL01 strain at different MOIs (1, 10 and 100; corresponding to Fp1, Fp10 and Fp100) or with *Escherichia coli* K12 LPS (100 ng/mL), used as a positive control. (*Upper left panel*) Heat map representing LEGENDplex analysis (N = 6), with blue referring to minimal induction and red to maximal induction. (*Other panels*) Cytokine concentrations and anti-inflammatory ratios for IL10, IL23, and TNF-α. MCP, monocyte chemoattractant protein. (*B*) Flow cytometry analysis of PBMCs stimulated for 16 hours in the same conditions as in panel *A*, showing $IL10^+$ cells among different immune cell populations (N = 5). DNeg, double negative $CD4^-CD8^-$ T-cell population. Data are mean ± standard error of the mean of 2 independent experiments. *$P < .05$, **$P < .01$, ***$P < .00,1$ and ****$P < .0001$, as determined by (*A*) mixed-effects analysis and Dunnett's multiple comparisons test and (*B*) ordinary 1-way analysis of variance.

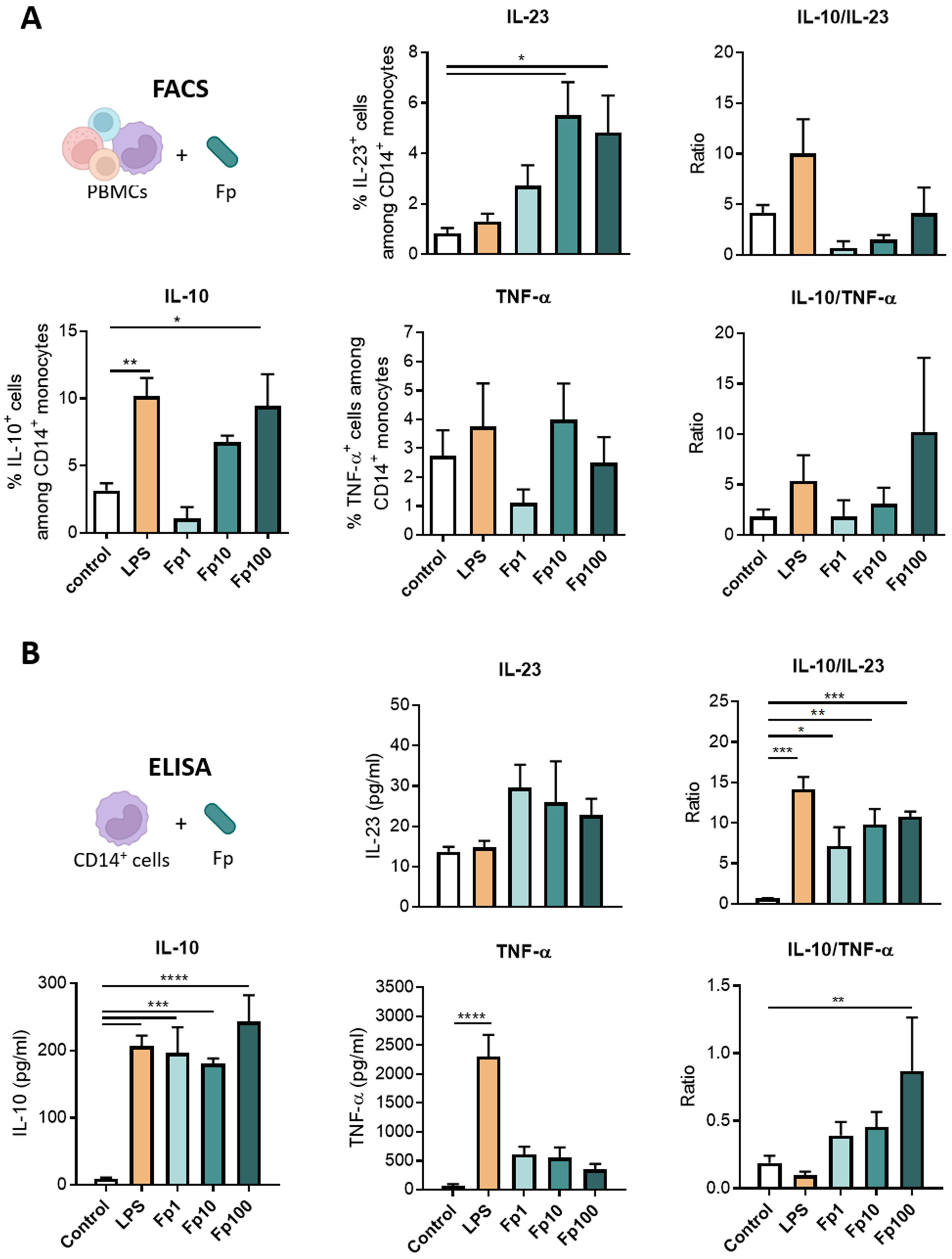

Figure 2. *F prausnitzii* EXL01 strain directly induces the production of IL10 in CD14$^+$ monocytes from systemic circulation. (*A*) Flow cytometry analysis of PBMCs stimulated in the same conditions as in Figure 1, showing IL10$^+$, IL23$^+$, and TNF-α^+ cells among CD14$^+$ monocytes and anti-inflammatory ratios (N = 4). FACS, fluorescence-activated cell sorter. (*B*) Cytokine concentrations and anti-inflammatory ratios for IL10, IL23, and TNF-α obtained by ELISA analysis (corrected by lactate dehydrogenase) of the supernatant of CD14$^+$ monocytes isolated from PBMCs and stimulated for 16 hours in the same conditions as in Figure 1 and 2*A*. Data are mean ± standard error of the mean of 2 independent experiments. **P* < .05, ***P* < .01, ****P* < .001, and *****P* < .0001, as determined by 1-way analysis of variance.

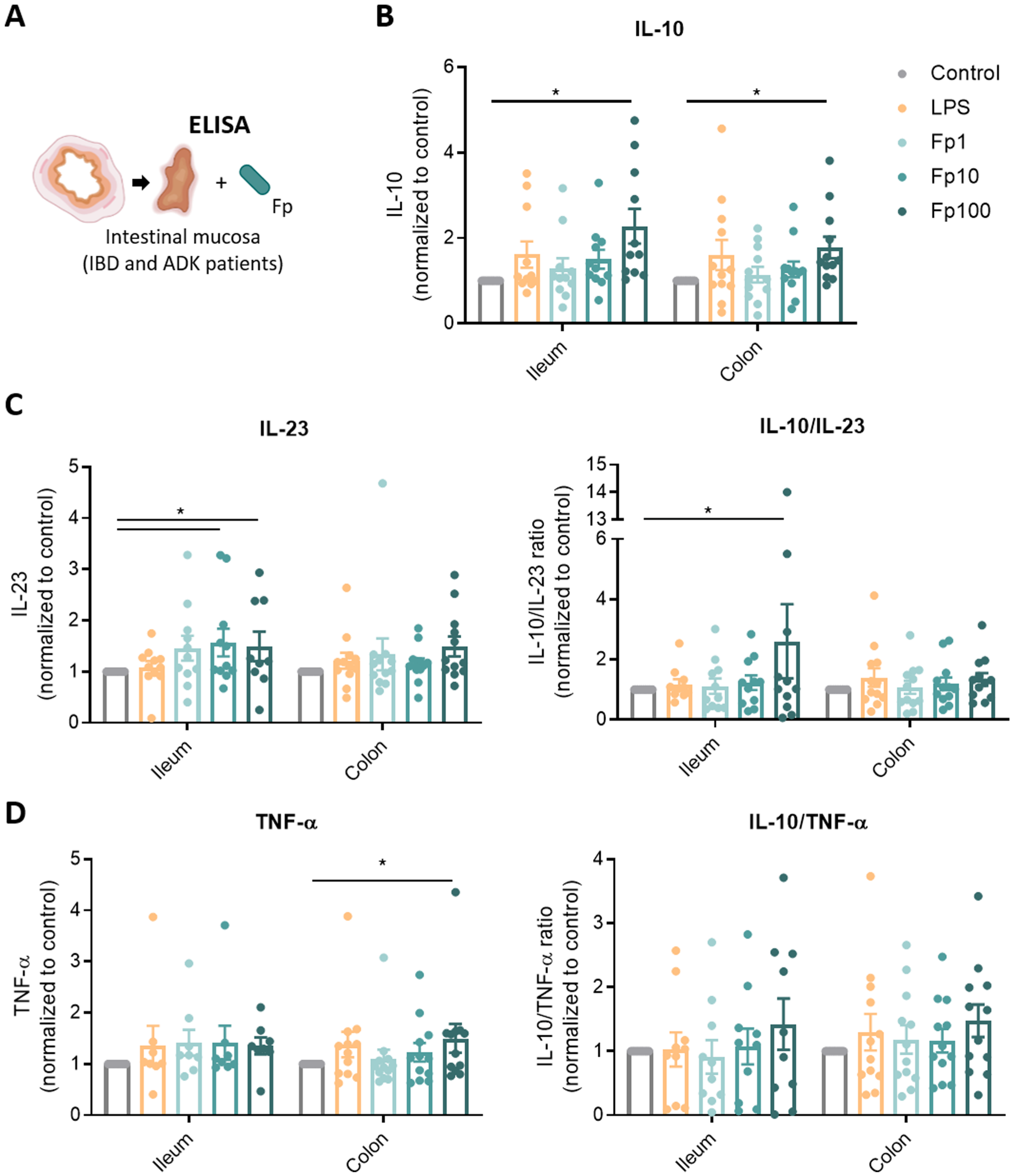

Figure 3. *F prausnitzii* induces the production of IL10 in intestinal tissue of IBD patients and noninflamed controls. (*A*) Schematic representation of the experiment setup. Concentrations of (*B*) IL10, (*C*) IL23, and (*D*) TNF-α and corresponding anti-inflammatory ratios measured by ELISA (corrected by lactate dehydrogenase) in the supernatant of intestinal mucosa (ileum and colon) from IBD and noninflamed controls (adenoma-carcinoma or ADK patients), stimulated for 16 hours with different doses of *F prausnitzii* EXL01 strain (Fp1, Fp10, Fp100) or LPS, as performed previously (N = 23: IBD ileum, N = 6; IBD colon, N = 8; ADK ileum, N = 5; ADK colon, N = 4). Data are mean ± standard error of the mean. *$P < .05$, as determined by 2-way analysis of variance.

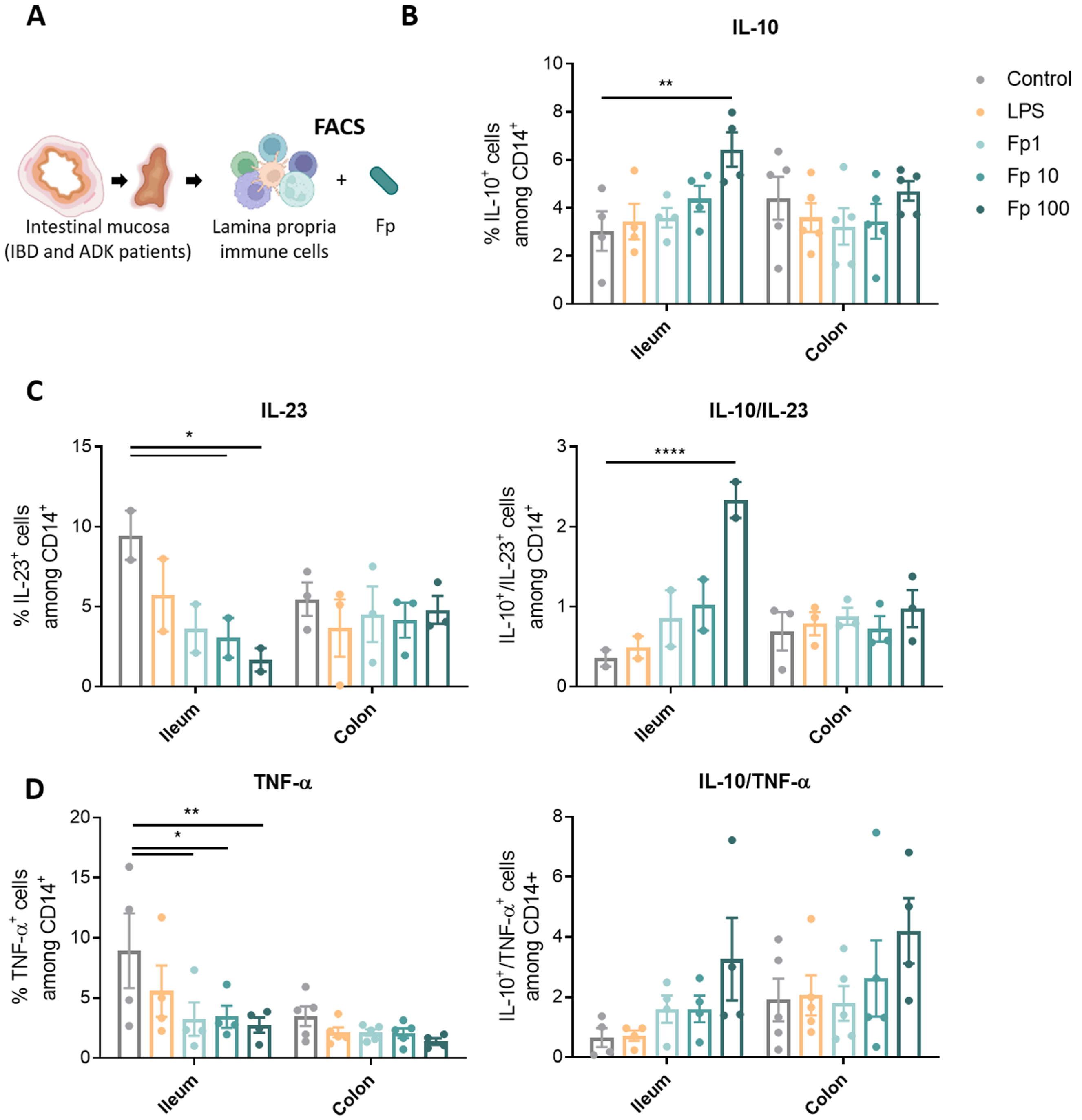

Figure 4. *F prausnitzii* induces the production of IL10 in CD14^{+} monocytes isolated from intestinal lamina propria of IBD patients and noninflamed controls. (*A*) Schematic representation of the experiment setup. FACS, fluorescence-activated cell sorter. Percentage of (*B*) IL10^{+}, (*C*) IL-23^{+}, and (*D*) TNF-α^{+} cells among CD14^{+} monocyte population measured by flow cytometry in the total lamina propria immune cells isolated from the intestinal mucosa (ileum and colon) of IBD and ADK patients, and stimulated for 16 hours with different doses of *F prausnitzii* EXL01 strain (Fp1, Fp10, Fp100) or LPS, as performed previously (N = 9: IBD ileum, N = 3; IBD colon, N = 2; ADK ileum, N = 1; ADK colon, N = 3). Data are mean ± standard error of the mean. *$P < .05$, **$P < .01$, and ****$P < .0001$ as determined by 2-way analysis of variance.

Interestingly, oligomycin reduced the IL10/TNF-α anti-inflammatory ratio induced by *F prausnitzii* EXL01 strain, but not by LPS, suggesting that the anti-inflammatory response induced by *F prausnitzii* EXL01 strain is dependent on mitochondrial respiration (Figure 6*A*). Real-time bioenergetic profile analysis using Seahorse technology showed that basal stimulation with *F prausnitzii* EXL01 strain, particularly Fp100, increased OXPHOS assessed through the oxygen consumption rate measurement (Figure 6*B* and Supplementary Figure 6*B*). Besides, Fp100

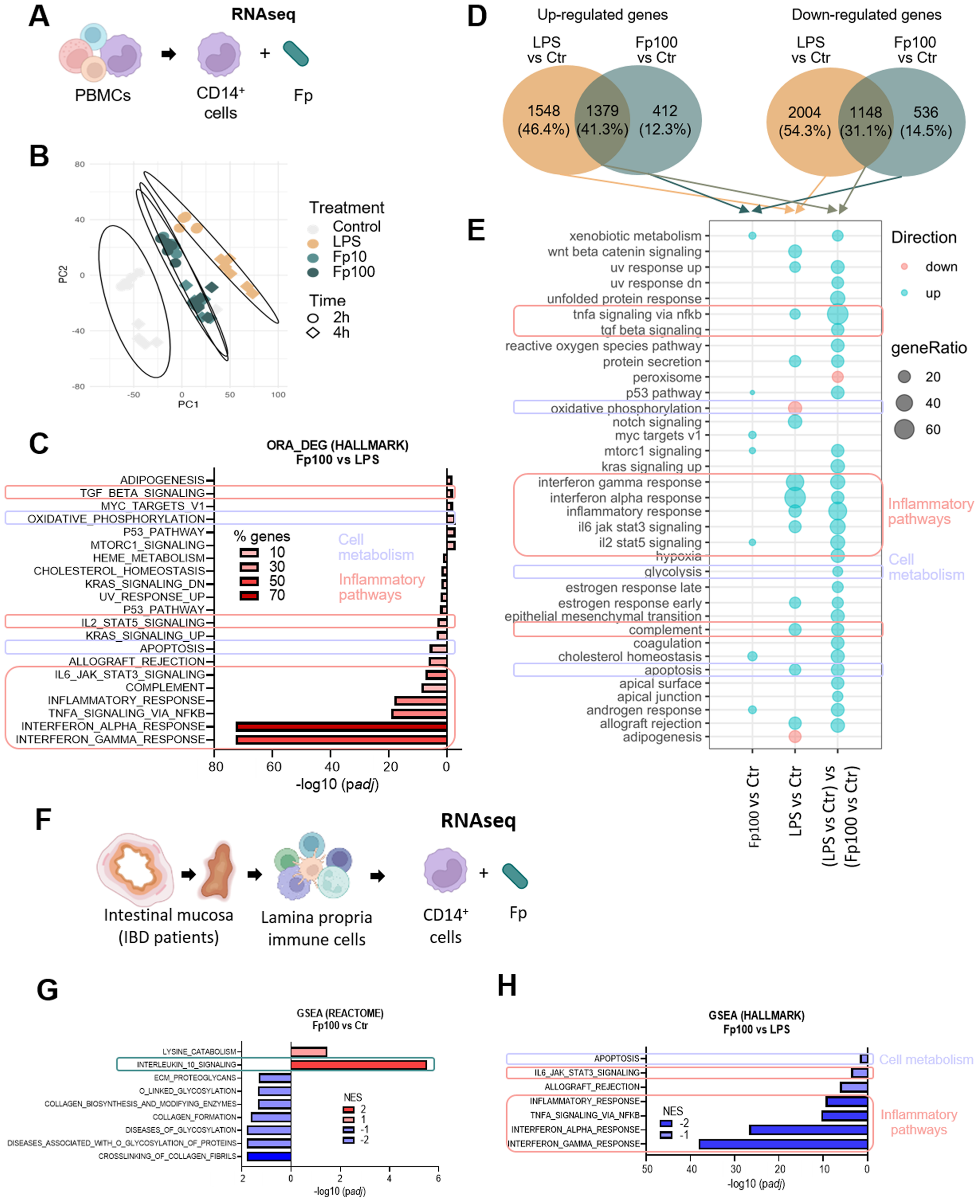
A
RNAseq
PBMCs
CD14+ cells
Fp
B
Treatment
Control
LPS
Fp10
Fp100
Time
2h
4h
PC1
PC2
C
ORA_DEG (HALLMARK)
Fp100 vs LPS
ADIPOGENESIS
TGF_BETA_SIGNALING
MYC_TARGETS_V1
OXIDATIVE_PHOSPHORYLATION
P53_PATHWAY
MTORC1_SIGNALING
HEME_METABOLISM
CHOLESTEROL_HOMEOSTASIS
KRAS_SIGNALING_DN
UV_RESPONSE_UP
P53_PATHWAY
IL2_STAT5_SIGNALING
KRAS_SIGNALING_UP
APOPTOSIS
ALLOGRAFT_REJECTION
IL6_JAK_STAT3_SIGNALING
COMPLEMENT
INFLAMMATORY_RESPONSE
TNFA_SIGNALING_VIA_NFKB
INTERFERON_ALPHA_RESPONSE
INTERFERON_GAMMA_RESPONSE
% genes
10
30
50
70
Cell metabolism
Inflammatory pathways
-log10 (padj)
D
Up-regulated genes
LPS vs Ctr
Fp100 vs Ctr
1548 (46.4%)
1379 (41.3%)
412 (12.3%)
Down-regulated genes
2004 (54.3%)
1148 (31.1%)
536 (14.5%)
E
xenobiotic metabolism
wnt beta catenin signaling
uv response up
uv response dn
unfolded protein response
tnfa signaling via nfkb
tgf beta signaling
reactive oxygen species pathway
protein secretion
peroxisome
p53 pathway
oxidative phosphorylation
notch signaling
myc targets v1
mtorc1 signaling
kras signaling up
interferon gamma response
interferon alpha response
inflammatory response
il6 jak stat3 signaling
il2 stat5 signaling
hypoxia
glycolysis
estrogen response late
estrogen response early
epithelial mesenchymal transition
complement
coagulation
cholesterol homeostasis
apoptosis
apical surface
apical junction
androgen response
allograft rejection
adipogenesis
Fp100 vs Ctr
LPS vs Ctr
(LPS vs Ctr) vs (Fp100 vs Ctr)
Direction
down
up
geneRatio
20
40
60
Inflammatory pathways
Cell metabolism
F
RNAseq
Intestinal mucosa (IBD patients)
Lamina propria immune cells
CD14+ cells
Fp
G
GSEA (REACTOME)
Fp100 vs Ctr
LYSINE_CATABOLISM
INTERLEUKIN_10_SIGNALING
ECM_PROTEOGLYCANS
O_LINKED_GLYCOSYLATION
COLLAGEN_BIOSYNTHESIS_AND_MODIFYING_ENZYMES
COLLAGEN_FORMATION
DISEASES_OF_GLYCOSYLATION
DISEASES_ASSOCIATED_WITH_O_GLYCOSYLATION_OF_PROTEINS
CROSSLINKING_OF_COLLAGEN_FIBRILS
NES
2
1
-1
-2
-log10 (padj)
H
GSEA (HALLMARK)
Fp100 vs LPS
APOPTOSIS
IL6_JAK_STAT3_SIGNALING
ALLOGRAFT_REJECTION
INFLAMMATORY_RESPONSE
TNFA_SIGNALING_VIA_NFKB
INTERFERON_ALPHA_RESPONSE
INTERFERON_GAMMA_RESPONSE
NES
-2
-1
Cell metabolism
Inflammatory pathways
-log10 (padj)

modulated oxygen consumption rate levels in response to oligomycin, FCCP, and rotenone and antimycin A, showing effects on OXPHOS parameters, including basal respiration, adenosine 5′-triphosphate (ATP) production, maximal respiration, and spare capacity of CD14$^+$ cells, contrary to LPS stimulation (Figure 6*B* and *C* and Supplementary Figure 6*B* and *C*).

On the other hand, LPS stimulation strongly decreased the above-mentioned parameters and increased nonmitochondrial respiration (corresponding to aerobic glycolysis), as previously demonstrated[38,39] (Figure 6*B* and *C*). Nonmitochondrial respiration is typically attributed to inflammation-associated enzymes, including cyclooxygenases, lipoxygenases, and reduced nicotinamide adenine dinucleotide phosphate oxidases, and increases with reactive oxygen species[40,41]; thus, it is considered as a negative indicator of the energetic health of the cell. Reciprocally, *F prausnitzii* EXL01 strain in basal conditions slightly decreased aerobic glycolysis in a dose-dependent manner, as measured by the extracellular acidification rate, whereas LPS stimulation increased this parameter (Figure 6*B*). Interestingly, increasing doses of *F prausnitzii* EXL01 strain blocked LPS-induced glycolysis activation and OXPHOS inhibition (Figure 6*B* and *C*).

Taken together, these results demonstrate that *F prausnitzii* EXL01 strain promotes IL10 production in monocytes without inducing the pro-inflammatory response triggered by LPS. Moreover, it is interesting to note that the functional changes in immune response are associated with more profound cellular metabolic modifications, as previously described in the case of other pro- or anti-inflammatory stimuli able to reprogram immune cell phenotypes and functions.[42]

To determine whether other bacteria have a similar effect, we stimulated CD14$^+$ cells isolated from fresh human PBMCs with LPS and/or EXL01, *C comes* 27758 strain (another anaerobic gram-positive Firmicutes thought to play a beneficial role in the gut), and *E coli* MG1655 strain and analyzed real-time bioenergetic profile using Seahorse technology and IL10 and TNF-α secretion by ELISA. In the absence or in the presence of LPS, *C comes* did not induce an increased OXPHOS or changes in OXPHOS parameters, including basal respiration, ATP production, maximal respiration, nonmitochondrial respiration, and spare capacity, of CD14$^+$ cells (Figure 7*A* and *B*), suggesting that this bacterium does not interfere with energy metabolism in CD14$^+$ monocytes. In the absence of LPS, *E coli* induced a decrease in OXPHOS and OXPHOS parameters, but no changes were observed in the presence of LPS (Figure 7*A* and *B*). In addition, EXL01 and *E coli* induced a significant increase in IL10 secretion compared with the control and to *C comes* (Figure 7*C*).

Concomitantly, the 3 bacteria induced increased TNF-α secretion, but to a much lower extent for EXL01, leading to a significantly higher IL10/TNF-α anti-inflammatory ratio for EXL01 (Figure 7*C*). In inflammatory conditions (when LPS was added), only EXL01 induced a significant increase of the IL10/TNF-α anti-inflammatory ratio (Figure 7*C*), indicating that the effect of EXL01 on energy metabolism in CD14$^+$ monocytes in an inflammatory context is associated with the induction of a regulatory phenotype.

Discussion

F prausnitzii is recognized as one of the most important bacteria of the human gut microbiome, and its anti-inflammatory properties make it a good candidate for the treatment of IBD. The anti-inflammatory effects of *F prausnitzii* are related to its ability to induce the production of IL10 by immune cells, but the cell types and the mechanisms underlying these effects, particularly in human settings, remain largely unknown. As previously described,[19] we confirmed that DP8 cells from human PBMCs produce IL10 in response to *F prausnitzii*. We identified CD14$^+$ monocytes as the primary IL10 producers in response to *F prausnitzii* in human PBMCs. *F prausnitzii* EXL01 strain can induce high IL10 production in a direct and dose-dependent manner in these cells, which is not associated with a

Figure 5. *F prausnitzii* EXL01 strain differentially affects immune response and cell energy metabolism compared with LPS in CD14$^+$ monocytes from peripheral human blood and from ileal human tissue. (*A*) Schematic representation of the analysis setup for CD14$^+$ monocytes from peripheral human blood (N = 6). (*B*) Principal component (PC) analysis showing the repartition of the expression profiles of blood CD14$^+$ monocytes according to their treatment; that is, different doses of *F prausnitzii* EXL01 strain (Fp10, Fp100) or LPS, as performed previously, for 2 and 4 hours. (*C*) Overrepresentation analysis of differentially expressed genes (DEGs) in the Fp100 compared with the LPS condition at 4 hours on Hallmark pathway database. *Left* are pathways enriched by down-regulated genes in Fp100 compared with LPS, *right* are pathways enriched by up-regulated genes. Percentages of the DEGs involved in each pathway are indicated by the red color intensity. (*D*) Venn's diagrams representing up- and down-regulated genes in Fp100 vs control (Ctr) conditions, LPS vs Ctr conditions, and between the LPS vs Ctr and Fp100 vs Ctr conditions. (*E*) Bubble plot showing significantly enriched pathways by up-regulated (in blue) and down-regulated (in red) genes in DEG lists. Bubble size shows the ratio of DEG genes involved in the pathways. Inflammatory pathways are encircled in pink, cell metabolism-related pathways in purple. (*F*) Schematic representation of the analysis setup for CD14$^+$ monocytes from lamina propria (N = 3). CD14$^+$ monocytes were isolated form the ileal lamina propria of IBD patients, and stimulated for 4 hours with different doses of *F prausnitzii* EXL01 strain (Fp10, Fp100) or LPS, as performed previously. (*G*) Significant enriched REACTOM pathways in the comparison between Fp100 and Ctr conditions at 4 hours identified by gene set enrichment analysis. Interleukin_10_Signaling pathway is encircled in green. (*H*) Significant enriched Hallmark pathways in the comparison between Fp100 and LPS conditions at 4 hours by gene set enrichment analysis. *Left/blue* are down-regulated pathways in Fp100 compared with Ctr or LPS conditions, *right/red* up-regulated pathways. NES, Normalized Enrichment Score. Inflammatory pathways are encircled in pink, cell metabolism-related pathways in purple.

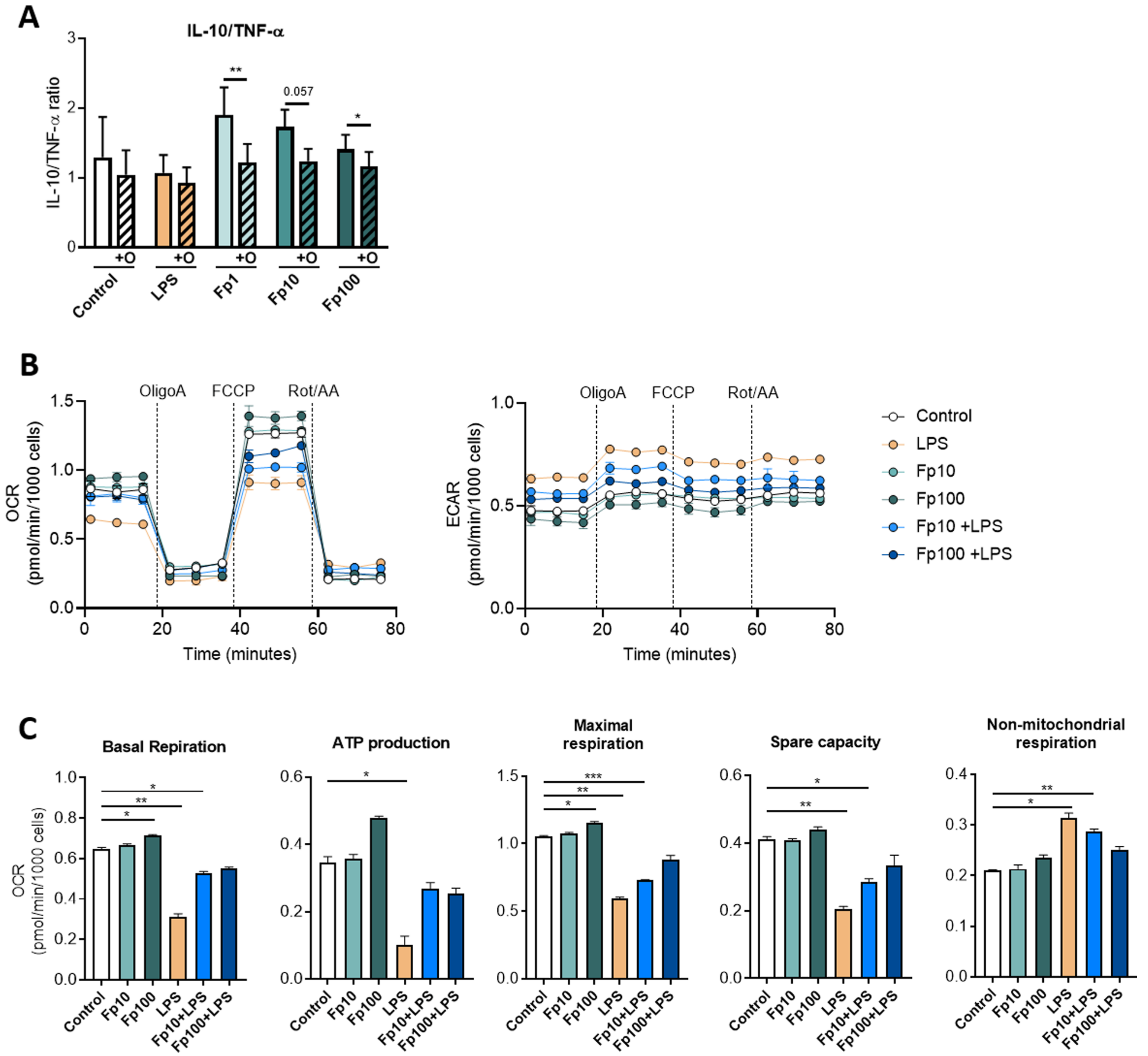

Figure 6. *F prausnitzii* EXL01 strain relies on mitochondrial activity to induce an anti-inflammatory response in CD14$^+$ monocytes. (*A*) IL10/TNF-α anti-inflammatory ratio as measured by ELISA in the supernatant of CD14$^+$ monocytes purified from PBMCs and stimulated for 16 hours as performed previously, except from an additional step of 1-hour pretreatment with oligomycin, an inhibitor of oxidative phosphorylation (ATP synthase). (*B*) Oxygen consumption rate (OCR) and extracellular acidification rate (ECAR) of CD14$^+$ blood monocytes stimulated for 6 hours with different doses of *F prausnitzii* EXL01 strain (Fp10 and Fp100) or LPS, as performed previously or with the combination of Fp10 or Fp100 and LPS (Fp10+LPS and Fp100+LPS), measured during a Seahorse Cell Mito Stress assay. Rot/AA, rotenone and antimycin A. (*C*) Basal respiration, ATP production, maximal respiration, spare capacity, and nonmitochondrial respiration obtained from the Seahorse Cell Mito Stress assay, as represented on the schematic OCR graph in Supplementary Figure 6 (N = 3). **P* < .05, ***P* < .01 and ****P* < .001, as determined by (*A*) paired *t* test or (*C*) mixed-effects analysis and Dunnett's multiple comparisons test.

pro-inflammatory response, as triggered by the bacterial factor LPS. This result was confirmed on CD14$^+$ monocytes isolated not only from fresh human blood but also from the lamina propria of ileum and colon mucosa of patients with IBD and noninflamed controls right after surgery, confirming also the wide distribution of IL10-producing immune cells in both ileum and colonic tissues.[43]

Using inhibitor and Seahorse technology, we found out that in parallel to immunomodulatory properties, *F prausnitzii* also induces profound changes in the energy metabolism of monocytes, with increased mitochondrial activity, in contrast to LPS and to *C comes* and *E coli*. Moreover, we showed that the anti-inflammatory response induced by *F prausnitzii* EXL01 strain is dependent on mitochondrial activity.

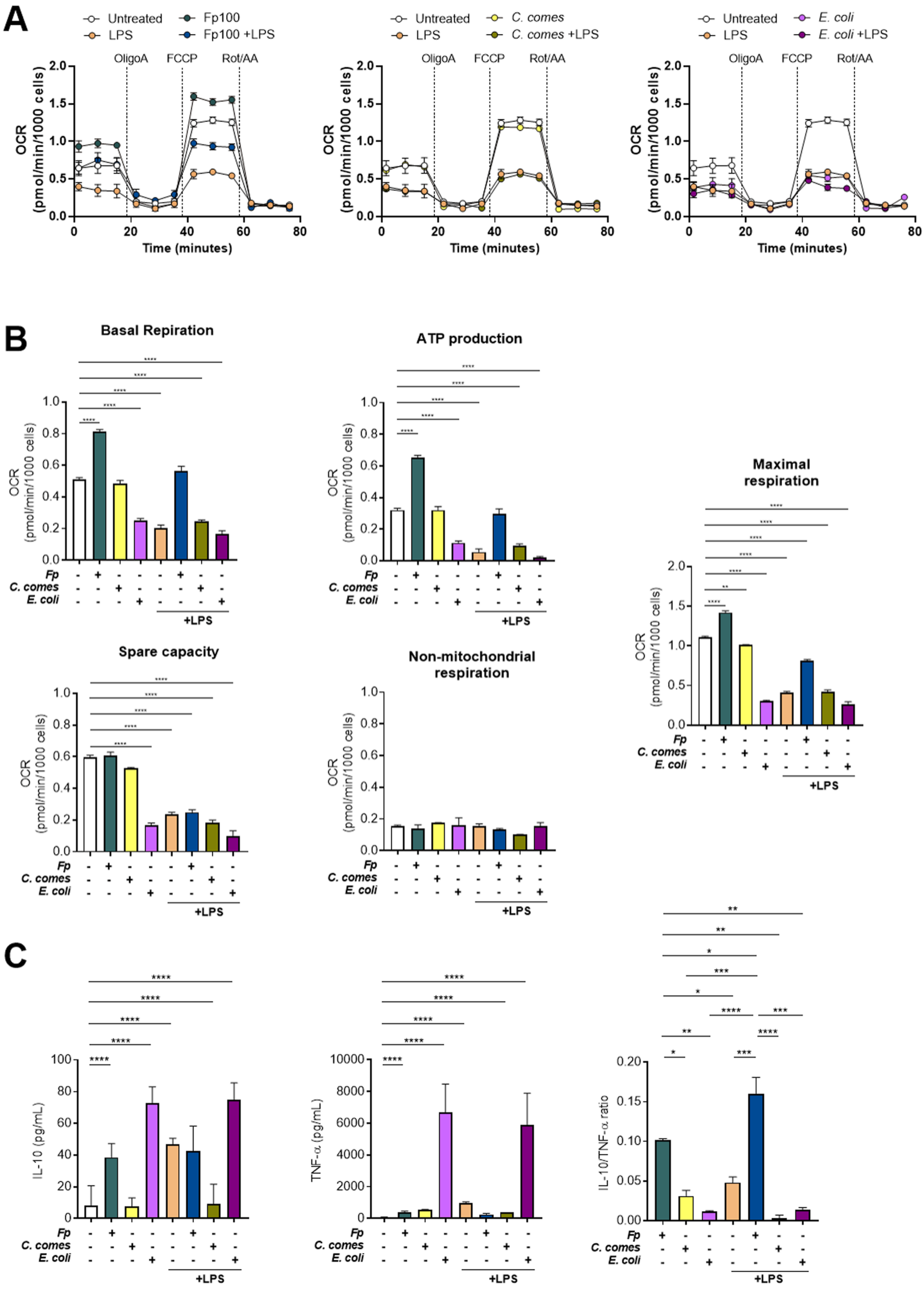

Figure 7. Anti-inflammatory effects of *F prausnitzii* EXL01 strain relies on rewiring energy metabolism in CD14$^+$ monocytes. (*A*) Oxygen consumption rate (OCR) of CD14$^+$ blood monocytes stimulated for 6 hours with *F prausnitzii* EXL01 strain (Fp100), *C comes*, *E coli*, or LPS as performed previously, in presence or not of LPS measured during a Seahorse Cell Mito Stress assay. (*B*) Basal respiration, ATP production, maximal respiration, spare capacity, and nonmitochondrial respiration obtained from the Seahorse Cell Mito Stress assay. (*C*) Cytokine concentrations and anti-inflammatory ratios for IL10 and TNF-α obtained by ELISA analysis (corrected by lactate dehydrogenase) of the supernatant of CD14+ monocytes stimulated as in panel *A* (N = 3). **P* < .05, ***P* < .01, ****P* < .001, *****P* <.0001 as determined by mixed-effects analysis and Dunnett's test correction for multiple comparisons.

Under environmental stimulation, immune cells undergo functional changes, from resting to activation state, which relies on reprogramming of their energy metabolism.[42] The main metabolic pathways involved in immune metabolism are glycolysis, the tricarboxylic acid (TCA) cycle, the pentose phosphate pathway, fatty acid oxidation and synthesis, and amino acid metabolism. For instance, macrophage immune functions are associated with their energy metabolism. In homeostasis, macrophages mostly use the TCA cycle, but their metabolism is modified when activated by the bacterial product LPS.[44,45]

Known as M1 or classically activated macrophages, LPS-activated macrophages display pro-inflammatory properties. Their TCA cycle is altered, and glucose uptake is enhanced and switches to glycolysis, which is associated with the activation of the pentose phosphate pathway for biosynthesis of biomolecules and ATP production. In contrast, IL4-activated macrophages, namely M2 or alternately activated macrophages, have anti-inflammatory and antiparasitic effects, and play roles in wound healing.[46] Moreover, M2 or alternately activated macrophages show different metabolic characteristics compared with M1, with glycolysis used to support OXPHOS, leading to enhanced fatty acid oxidation and OXPHOS.[46] Our results suggest that the *F prausnitzii* EXL01 strain reprograms host intestinal immune cells toward an alternatively activated phenotype with increased mitochondrial activity, although the precise mechanisms and dialogue involved remain to be elucidated.

The gut microbiota is a major actor in the modulation of both immunity and cellular energy metabolism. Despite the crucial role of cellular energy metabolism in the ability to mount an appropriate immune response, the interactions between microbiota and immune cells regulation remain poorly understood. Most studies have focused on surface polysaccharides, including LPS, and in microbiota-derived molecules, either produced or transformed by microorganisms, such as short-chain fatty acids, tryptophan metabolites, lipids, and bile acids.[7,47–51] We and others showed that *Faecalibacterium* exhibits anti-inflammatory effects both in vitro and in vivo in colitis models, with several mechanisms of action, including the microbial anti-inflammatory molecule–secreted peptides, cell wall components, butyrate production, and extracellular vesicles.[23,52,53] However, the bacterial molecules, the host receptors, and signaling pathways involved are still poorly understood and require further investigations. Here, we should remind that *F prausnitzii* is an extremely oxygen-sensitive bacterium. Even brief exposure to oxygen can hinder its growth, as well as its ability to regrow when returned to strictly anaerobic conditions. In both in vitro and in vivo experiments, oxygen exposure results in the loss of *F prausnitzii* culturability. However, despite this, in our study, along with previous research,[19,20] the bacterium still exhibits potent biological effects, suggesting that the biological activity of *F prausnitzii* does not necessarily depend on metabolically active or culturable bacteria. The identification of bacterial molecules, host receptors, and signaling pathways involved in the anti-inflammatory properties of *F prausnitzii* will be the subject of future research.

Studies of immune cell energy metabolism revealed the metabolic mechanisms underlying disease progression, notably inflammatory diseases such as autoimmune diseases, chronic viral infections, and cancer.[42] In the case of IBD, a link has been found between mitochondrial dysfunction and disease severity, mostly in intestinal epithelial cells.[50,54–58] It is thus crucial to decipher more precisely the impact of host-microbiota interactions in health and disease, especially in immune and metabolic regulation. The intrinsic diversity of the actors within the gut microbiota and the immune system brings an additional level of difficulty in the exploration of this cross talk. However, this effort is crucial to identify innovative therapeutic targets for microbiota-associated diseases.

Thus, the *F prausnitzii* EXL01 strain, well characterized and already in the clinical development stage for IBD (NCT05542355 and NCT06925061), is an ideal candidate strain for the development of live biotherapeutic products in the context of intestinal inflammation. Oral administration of an *F prausnitzii*-based product could alleviate IBD patient symptoms and lead to more extended remission periods.

Supplementary Material

Note: To access the supplementary material accompanying this article, visit the online version of *Gastroenterology* at www.gastrojournal.org, and at https://doi.org/10.1053/j.gastro.2025.10.003.

References

1. Molodecky NA, Soon IS, Rabi DM, et al. Increasing incidence and prevalence of the inflammatory bowel diseases with time, based on systematic review. Gastroenterology 2012;142:46–54.e42; quiz: e30.
2. Goethel A, Croitoru K, Philpott DJ. The interplay between microbes and the immune response in inflammatory bowel disease. J Physiol 2018;596:3869–3882.
3. Jostins L, Ripke S, Weersma RK, et al. Host-microbe interactions have shaped the genetic architecture of inflammatory bowel disease. Nature 2012;491:119–124.
4. Khor B, Gardet A, Xavier RJ. Genetics and pathogenesis of inflammatory bowel disease. Nature 2011;474:307–317.
5. Lamas B, Richard ML, Leducq V, et al. CARD9 impacts colitis by altering gut microbiota metabolism of tryptophan into aryl hydrocarbon receptor ligands. Nat Med 2016;22:598–605.
6. Liu JZ, van Sommeren S, Huang H, et al. Association analyses identify 38 susceptibility loci for inflammatory bowel disease and highlight shared genetic risk across populations. Nat Genet 2015;47:979–986.

7. Lavelle A, Sokol H. Gut microbiota–derived metabolites as key actors in inflammatory bowel disease. Nat Rev Gastroenterol Hepatol 2020;17:223–237.
8. Nishida A, Inoue R, Inatomi O, et al. Gut microbiota in the pathogenesis of inflammatory bowel disease. Clin J Gastroenterol 2018;11:1–10.
9. Britton GJ, Contijoch EJ, Mogno I, et al. Microbiotas from humans with inflammatory bowel disease alter the balance of gut Th17 and RORgammat(+) regulatory T cells and exacerbate colitis in mice. Immunity 2019;50:212–224.e4.
10. Benech N, Sokol H. Fecal microbiota transplantation in gastrointestinal disorders: time for precision medicine. Genome Med 2020;12:58.
11. Sokol H, Landman C, Seksik P, et al. Fecal microbiota transplantation to maintain remission in Crohn's disease: a pilot randomized controlled study. Microbiome 2020;8:12.
12. Gevers D, Kugathasan S, Denson LA, et al. The treatment-naive microbiome in new-onset Crohn's disease. Cell Host Microbe 2014;15:382–392.
13. Lloyd-Price J, Arze C, Ananthakrishnan AN, et al. Multi-omics of the gut microbial ecosystem in inflammatory bowel diseases. Nature 2019;569:655–662.
14. Sokol H, Seksik P, Furet JP, et al. Low counts of *Faecalibacterium prausnitzii* in colitis microbiota. Inflamm Bowel Dis 2009;15:1183–1189.
15. Hold GL, Schwiertz A, Aminov RI, et al. Oligonucleotide probes that detect quantitatively significant groups of butyrate-producing bacteria in human feces. Appl Environ Microbiol 2003;69:4320–4324.
16. Lopez-Siles M, Duncan SH, Garcia-Gil LJ, et al. *Faecalibacterium prausnitzii*: from microbiology to diagnostics and prognostics. ISME J 2017;11:841–852.
17. Miquel S, Martin R, Rossi O, et al. *Faecalibacterium prausnitzii* and human intestinal health. Curr Opin Microbiol 2013;16:255–261.
18. Sokol H, Pigneur B, Watterlot L, et al. *Faecalibacterium prausnitzii* is an anti-inflammatory commensal bacterium identified by gut microbiota analysis of Crohn disease patients. Proc Natl Acad Sci U S A 2008;105:16731–16736.
19. Touch S, Godefroy E, Rolhion N, et al. Human CD4+CD8alpha+ Tregs induced by *Faecalibacterium prausnitzii* protect against intestinal inflammation. JCI Insight 2022;7:e154722.
20. Bredon M, Danne C, Pham HP, et al. *Faecalibaterium prausnitzii* strain EXL01 boosts efficacy of immune checkpoint inhibitors. Oncoimmunology 2024;13:2374954.
21. Dikeocha IJ, Al-Kabsi AM, Chiu HT, et al. *Faecalibacterium prausnitzii* ameliorates colorectal tumorigenesis and suppresses proliferation of HCT116 colorectal cancer cells. Biomedicines 2022;10:1128.
22. Martin R, Miquel S, Chain F, et al. *Faecalibacterium prausnitzii* prevents physiological damages in a chronic low-grade inflammation murine model. BMC Microbiol 2015;15:67.
23. Quevrain E, Maubert MA, Michon C, et al. Identification of an anti-inflammatory protein from *Faecalibacterium prausnitzii*, a commensal bacterium deficient in Crohn's disease. Gut 2016;65:415–425.
24. Zhang M, Qiu X, Zhang H, et al. *Faecalibacterium prausnitzii* inhibits interleukin-17 to ameliorate colorectal colitis in rats. PLoS One 2014;9:e109146.
25. Qiu X, Zhang M, Yang X, et al. *Faecalibacterium prausnitzii* upregulates regulatory T cells and anti-inflammatory cytokines in treating TNBS-induced colitis. J Crohns Colitis 2013;7:e558–e568.
26. Rossi O, van Berkel LA, Chain F, et al. *Faecalibacterium prausnitzii* A2-165 has a high capacity to induce IL-10 in human and murine dendritic cells and modulates T cell responses. Sci Rep 2016;6:18507.
27. Alameddine J, Godefroy E, Papargyris L, et al. *Faecalibacterium prausnitzii* skews human DC to prime IL10-producing T cells through TLR2/6/JNK signaling and IL-10, IL-27, CD39, and IDO-1 Induction. Front Immunol 2019;10:143.
28. Sarrabayrouse G, Bossard C, Chauvin JM, et al. CD4CD8alphaalpha lymphocytes, a novel human regulatory T cell subset induced by colonic bacteria and deficient in patients with inflammatory bowel disease. PLoS Biol 2014;12:e1001833.
29. Krueger F, James F, Ewels P, et al. FelixKrueger/TrimGalore: v0.6.10—add default decompression path. Available at: https://zenodo.org/records/7598955. Published February 2, 2023.
30. Patro R, Duggal G, Love MI, et al. Salmon provides fast and bias-aware quantification of transcript expression. Nat Methods 2017;14:417–419.
31. Frankish A, Carbonell-Sala S, Diekhans M, et al. GENCODE: reference annotation for the human and mouse genomes in 2023. Nucleic Acids Res 2023;51:D942–D949.
32. Soneson C, Love MI, Robinson M. Differential analyses for RNA-seq: transcript-level estimates improve gene-level inferences. F1000Res 2016;4:1521.
33. Love MI, Huber W, Anders S. Moderated estimation of fold change and dispersion for RNA-seq data with DESeq2. Genome Biol 2014;15:550.
34. Leng N, Dawson JA, Thomson JA, et al. EBSeq: an empirical Bayes hierarchical model for inference in RNA-seq experiments. Bioinformatics 2013;29:1035–1043.
35. Li D, Zand MS, Dye TD, et al. An evaluation of RNA-seq differential analysis methods. PLoS One 2022;17:e0264246.
36. Korotkevich G, Sukhov V, Sergushichev A. Fast gene set enrichment analysis. bioRxiv 060012 February 1, 2021. https://doi.org/10.1101/060012 [preprint].
37. Subramanian A, Tamayo P, Mootha VK, et al. Gene set enrichment analysis: a knowledge-based approach for interpreting genome-wide expression profiles. Proc Natl Acad Sci U S A 2005;102:15545–15550.
38. Krawczyk CM, Holowka T, Sun J, et al. Toll-like receptor-induced changes in glycolytic metabolism regulate dendritic cell activation. Blood 2010;115:4742–4749.
39. Lee MKS, Al-Sharea A, Shihata WA, et al. Glycolysis is required for LPS-induced activation and adhesion of human CD14(+)CD16(-) monocytes. Front Immunol 2019;10:2054.

40. Chacko BK, Kramer PA, Ravi S, et al. The Bioenergetic Health Index: a new concept in mitochondrial translational research. Clin Sci (Lond) 2014;127:367–373.
41. Hill BG, Benavides GA, Lancaster JR Jr, et al. Integration of cellular bioenergetics with mitochondrial quality control and autophagy. Biol Chem 2012;393:1485–1512.
42. Hu C, Xuan Y, Zhang X, et al. Immune cell metabolism and metabolic reprogramming. Mol Biol Rep 2022;49:9783–9795.
43. Nguyen HD, Aljamaei HM, Stadnyk AW. The production and function of endogenous interleukin-10 in intestinal epithelial cells and gut homeostasis. Cell Mol Gastroenterol Hepatol 2021;12:1343–1352.
44. Rodriguez-Prados JC, Traves PG, Cuenca J, et al. Substrate fate in activated macrophages: a comparison between innate, classic, and alternative activation. J Immunol 2010;185:605–614.
45. Van den Bossche J, O'Neill LA, Menon D. Macrophage immunometabolism: where are we (going)? Trends Immunol 2017;38:395–406.
46. Caputa G, Flachsmann LJ, Cameron AM. Macrophage metabolism: a wound-healing perspective. Immunol Cell Biol 2019;97:268–278.
47. Backhed F, Ding H, Wang T, et al. The gut microbiota as an environmental factor that regulates fat storage. Proc Natl Acad Sci U S A 2004;101:15718–15723.
48. Cavallari JF, Barra NG, Foley KP, et al. Postbiotics for NOD2 require nonhematopoietic RIPK2 to improve blood glucose and metabolic inflammation in mice. Am J Physiol Endocrinol Metab 2020;318:E579–E585.
49. Honda K, Littman DR. The microbiota in adaptive immune homeostasis and disease. Nature 2016;535:75–84.
50. Michaudel C, Sokol H. The gut microbiota at the service of immunometabolism. Cell Metab 2020;32:514–523.
51. Rooks MG, Garrett WS. Gut microbiota, metabolites and host immunity. Nat Rev Immunol 2016;16:341–352.
52. Lenoir M, Martin R, Torres-Maravilla E, et al. Butyrate mediates anti-inflammatory effects of *Faecalibacterium prausnitzii* in intestinal epithelial cells through Dact3. Gut Microbes 2020;12:1–16.
53. Pan Y, Zhao X, Chen Q, et al. *Faecalibacterium prausnitzii* extracellular vesicles regulating macrophage differentiation via homologous recombination repair in colitis model. Microbiol Res 2025;298:128217.
54. Hsieh SY, Shih TC, Yeh CY, et al. Comparative proteomic studies on the pathogenesis of human ulcerative colitis. Proteomics 2006;6:5322–5331.
55. Khaloian S, Rath E, Hammoudi N, et al. Mitochondrial impairment drives intestinal stem cell transition into dysfunctional Paneth cells predicting Crohn's disease recurrence. Gut 2020;69:1939–1951.
56. Liu TC, Gurram B, Baldridge MT, et al. Paneth cell defects in Crohn's disease patients promote dysbiosis. JCI Insight 2016;1:e86907.
57. Mottawea W, Chiang CK, Muhlbauer M, et al. Altered intestinal microbiota-host mitochondria crosstalk in new onset Crohn's disease. Nat Commun 2016;7:13419.
58. Theiss AL, Idell RD, Srinivasan S, et al. Prohibitin protects against oxidative stress in intestinal epithelial cells. FASEB J 2007;21:197–206.

Received October 8, 2024. Accepted October 1, 2025.

Correspondence
Address correspondence to: Nathalie Rolhion, PhD, CRSA, Hôpital Saint-Antoine, 27 rue de Chaligny, 75012 Paris, France. e-mail: nathalie.rolhion@inserm.fr; or Harry Sokol, MD, PhD, Service de Gastro-entérologie, Hôpital Saint-Antoine, 184 rue du Faubourg Saint-Antoine, 75571 Paris Cedex 12, France. e-mail: harry.sokol@gmail.com.

Acknowledgments
The authors thank the healthy volunteers, the patients recruited in the Digestive Surgery Department of Saint-Antoine Hospital (Assistance publique-Hôpitaux de Paris) and the nurses; Sandrine Truong (Gastroenterology Department of Saint-Antoine Hospital); URC-Est (AP-HP) for its logistics for the research; Professor Mohamad Mohty and Dr Béatrice Gaugler (Saint-Antoine Research Center, Paris) for the use of the CytoFLEX flow cytometer; Yannick Marie, Delphine Bouteiller, and Emeline Mundwiller (Platform ICM Paris Brain Institute) for RNA sequencing; Delphine Scalbert-Sterlin (Sorbonne Université, Paris) for the gift of the *Coprococcus comes* strain, and Benjamin Hadida (Exeliom Biosciences) for helpful discussion.

CRediT Authorship Contributions
Nathalie Rolhion, PhD (Conceptualization: Equal; Funding acquisition: Equal; Investigation: Equal; Methodology: Equal; Supervision: Equal; Validation: Equal; Writing – review & editing: Equal)

Camille Danne, PhD (Conceptualization: Equal; Data curation: Lead; Formal analysis: Lead; Investigation: Equal; Writing – original draft: Lead; Writing – review & editing: Equal)

Laura Creusot, MS (Investigation: Supporting; Writing – review & editing: Supporting)

Rodrigo de Oliveira Formiga, PhD (Formal analysis: Supporting; Investigation: Supporting; Methodology: Supporting; Writing – review & editing: Supporting)

Florian Marquet, MS (Formal analysis: Supporting; Investigation: Supporting; Methodology: Supporting; Writing – review & editing: Supporting)

Delphine Sedda, MS (Investigation: Supporting; Writing – review & editing: Supporting)

Laura Hua, BS (Investigation: Supporting; Writing – review & editing: Supporting)

Pauline Ruffié, MS (Conceptualization: Supporting; Funding acquisition: Supporting; Project administration: Equal; Resources: Supporting; Writing – review & editing: Supporting)

Hang-Phuong Pham, PhD (Data curation: Equal; Formal analysis: Equal; Methodology: Equal; Writing – review & editing: Supporting)

Iria Alonso Salgueiro, BS (Investigation: Supporting; Writing – review & editing: Supporting)

Loic Brot, BS (Investigation: Supporting; Writing – review & editing: Supporting)

Marie-Laure Michel, PhD (Investigation: Supporting; Writing – review & editing: Supporting)

Philippe Langella, PhD (Writing – review & editing: Supporting)

Jérémie H. Lefevre, MD (Resources: Equal; Writing – review & editing: Supporting)

Harry Sokol, MD, PhD (Conceptualization: Equal; Funding acquisition: Equal; Investigation: Equal; Methodology: Equal; Supervision: Equal; Writing – review & editing: Equal)

Conflicts of interest
These authors disclose the following: Harry Sokol reports lecture fees, board membership, or consultancy from Carenity, AbbVie, Astellas, Danone, Ferring, Mayoly Spindler, MSD, Novartis, Roche, Tillots, Enterome, BiomX, Takeda, and Biocodex, has stocks from Enterome, and is co-founder of Exeliom Biosciences. Philippe Langella reports lecture fees, board membership, or consultancy from Biose, Biostime, Boiron, Bonduelle, BMS, Bromatech, IPSEN, iTaK, Lallemand, Lesaffre, L'Oréal, Mayoli, Merck, Procter and Gamble, Second Genome, Therascience, and URGO, and is co-founder of Exeliom Biosciences. Jérémie H. Lefevre reports lecture fees from Ethicon, Takeda, Intuitive, and B-Braun, invitation to a medical congress by Biomup, Intuitive, and MD Start, is a consultant for Safeheal, Coloplast, and FSK, and is a personal investor in 1 digital companies, medical device companies or biotech companies. Delphine Sedda, Laura Hua, and Pauline Ruffié are employees of Exeliom Biosciences. The remaining authors disclose no conflicts.

Funding
The original study design was initially discussed between the research group and Exeliom Biosciences. This study received funding from Exeliom Biosciences, European Research Council (ERC, ENERGISED, ERC-2021-COG-101043802) and MSD-AVENIR.

Data Availability
RNA-sequencing data reported in this paper have been deposited in GEO (#GSE285037 (PBMC) and #GSE285449 (ileal human tissue).

Gastroenterology, Volume *170*

Supplemental information

Faecalibacterium prausnitzii Induces an Anti-inflammatory Response and a Metabolic Reprogramming in Human Monocytes

Camille Danne, Rodrigo de Oliveira Formiga, Laura Creusot, Florian Marquet, Delphine Sedda, Laura Hua, Pauline Ruffié, Hang-Phuong Pham, Loic Brot, Iria Alonso Salgueiro, Marie-Laure Michel, Philippe Langella, Jérémie H. Lefevre, Harry Sokol, and Nathalie Rolhion

Supplementary figure and table legends

Supplementary figure 1. (A) Cytokine concentrations and anti-inflammatory ratios for IL-10 and IL-23 obtained by ELISA analysis (corrected by LDH) in the supernatant of $CD14^+$ monocytes isolated from PBMCs and stimulated in the same conditions as in Fig.1 and 2 (N=8). *P<.05, **P<.01 and ***P<.001 as determined by mixed-effects analysis and Dunnett's multiple comparisons test. (B) Gating strategy for flow cytometry analysis of the different immune cell populations in PBMCs.

Supplementary figure 2. (A) Gating strategy for flow cytometry analysis of monocytes in PBMCs. (B) FACS plot representing CD14 and CD16 expression in PBMCs stimulated by different doses of *F. prausnitzii* EXL01 strain (Fp1, Fp10, Fp100) or LPS. (C) Percentage of classical ($CD14^+CD16^-$), intermediate ($CD14^+CD16^+$), non-classical ($CD14^-CD16^+$) and total $CD14^+$ monocytes among live stimulated PBMCs. (D) FACS plot representing the percentage of $CD14^+$ cells before and after isolation of $CD14^+$ cells from PBMCs using magnetic beads.

Supplementary figure 3. IL-10 concentrations measured by ELISA (corrected by LDH) in the supernatant of intestinal mucosa (ileum and colon) from non-inflamed controls (adenoma-carcinoma or ADK patients) and IBD patients showed separately, stimulated for 16 h with different doses of *F. prausnitzii* EXL01 strain (Fp1, Fp10, Fp100) or LPS, as performed previously. N=23 (IBD ileum N=6; IBD colon N=8; ADK ileum N=5; ADK colon N=4). Data are mean ± SEM.

Supplementary figure 4. (A) Gating strategy for flow cytometry analysis of IL-10^+ monocytes in the lamina propria immune cells isolated from intestinal mucosa. (B) Percentage of IL-10^+ cells among $CD14^+$ monocytes in the lamina propria immune cells isolated from intestinal mucosa (ileum and colon) from ADK and IBD patients showed separately, stimulated for 16 h with different doses of *F. prausnitzii*

EXL01 strain (Fp1, Fp10, Fp100) or LPS, as performed previously. N=9 (IBD ileum N=3; IBD colon N=2; ADK ileum N=1; ADK colon N=3). (C) Percentage of IL-10$^+$ cells among CD3$^+$ T and CD19$^+$ B cell populations in the lamina propria immune cells isolated from intestinal mucosa (ileum and colon) from IBD and ADK patients, stimulated for 16 h, as performed previously. Data are mean ± SEM.

Supplementary figure 5. Over-representation analysis of the differential gene expression profiles of PBMCs between the Fp100 and Ctr conditions (A), LPS and Ctr conditions (B) and Fp100 and LPS conditions (C) at 4 h on REACTOME pathway database. Interleukin_10_Signaling pathway is encircled in green, inflammatory pathways in pink and cell metabolism-related pathways in purple.

Supplementary figure 6. (A) Schematic representation of the two main energy metabolic pathways, glycolysis and mitochondrial respiration, including TCA cycle and oxidative phosphorylation. TCA, tricarboxylic acid cycle or Krebs cycle; ADP, adenine diphosphate; ATP, adenine triphosphate; NADH, Nicotinamide adenine dinucleotide; FADH2, *Flavin adenine dinucleotide.* (B) Schematic representation of the mitochondrial respiratory chain with the targets of each inhibitor used in the Seahorse Mito Stress assay. (C) Schematic representation of Oxygen Consumption Rate (OCR) evolution in time during a Mito Stress assay, showing basal respiration, ATP production, maximal respiration, spare capacity and non-mitochondrial respiration.

Suppl. Table 1. Characteristics of healthy individuals, controls and IBD patients recruited in the study. Continuous variables are presented as mean (SD). Categorical variables are presented as counts (%). Control: Healthy margin of patients operated on for colon adenocarcinoma. IBD treatment for ELISA experiments: 5-ASA, hydrocortisone, prednisone, tofacitinib; for flow cytometry experiments: 5-ASA, prednisone, azathioprine, adalimumab, tofacitinib and for RNAseq experiments: ustekinumab,

filgotinib. Abbreviation: IBD, inflammatory bowel disease; CD, Crohn disease; UC, ulcerative colitis; SD, standard deviation; NA, not applicable; BMI, body mass index.

	PBMC and CD14+ cells	ELISA on total mucosa		Flow cytometry on lamina propria		RNAseq on lamina propria $CD14^{+}$ cells
	Healthy subjects	IBD	Control	IBD	Control	IBD
n	28	8	8	3	4	3
UC/CD	NA	7/1	NA	2/1	NA	2/1
Sex [Male]	18 (64.3%)	4 (50%)	2 (25%)	2 (66.7%)	3 (75%)	2 (66.7%)
Age [years]	35.9 (11.2)	48.1 (16.1)	60.5 (21.2)	41.3 (11.2)	65.6 (34.6)	49.3 (26.8)
BMI [kg/m^2]	24.2 (5.3)	25 (7)	26.1 (8.2)	26.9 (12)	24.5 (3.4)	24.4 (1.9)
Patient with IBD treatment at time of surgery	NA	4 (50%)	NA	2 (66.7%)	NA	2 (66.7%)
Type of tissue analyzed						
Colon	NA	3	6	2	3	NA
Ileum	NA	7	2	2	1	3

Suppl. Table 1.

Suppl Fig 1

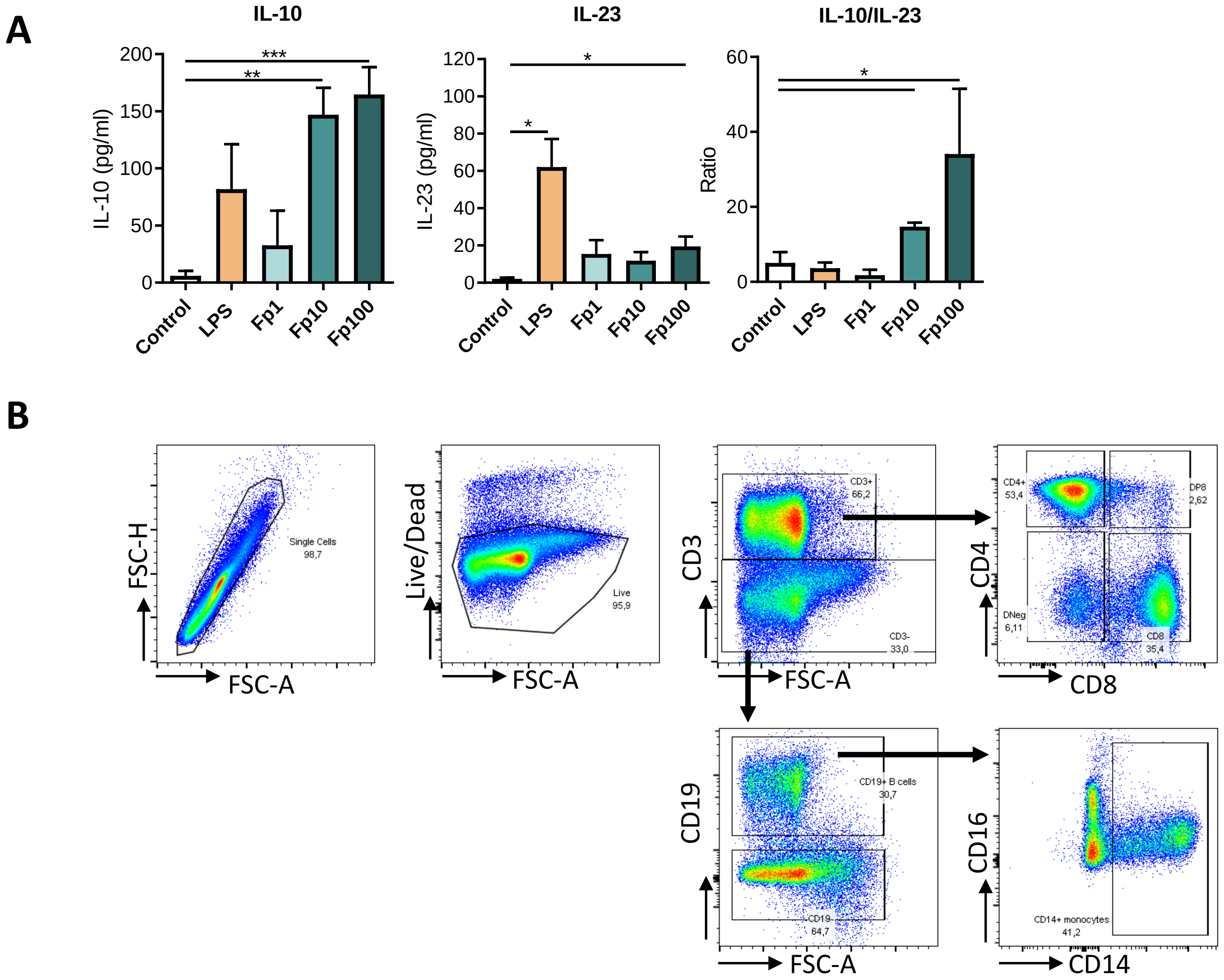
A
IL-10
IL-10 (pg/ml)
200
150
100
50
0
***
**
Control
LPS
Fp1
Fp10
Fp100
IL-23
IL-23 (pg/ml)
120
100
80
60
40
20
0
*
*
IL-10/IL-23
Ratio
60
40
20
0
*
B
FSC-H
FSC-A
Single Cells
98,7
Live/Dead
Live
95,9
CD3
CD3+
66,2
CD3-
33,0
CD4
CD8
CD4+
53,4
DP8
2,62
DNeg
6,11
CD8
35,4
CD19
CD19+ B cells
30,7
CD19-
64,7
CD16
CD14
CD14+ monocytes
41,2

Suppl Fig 2

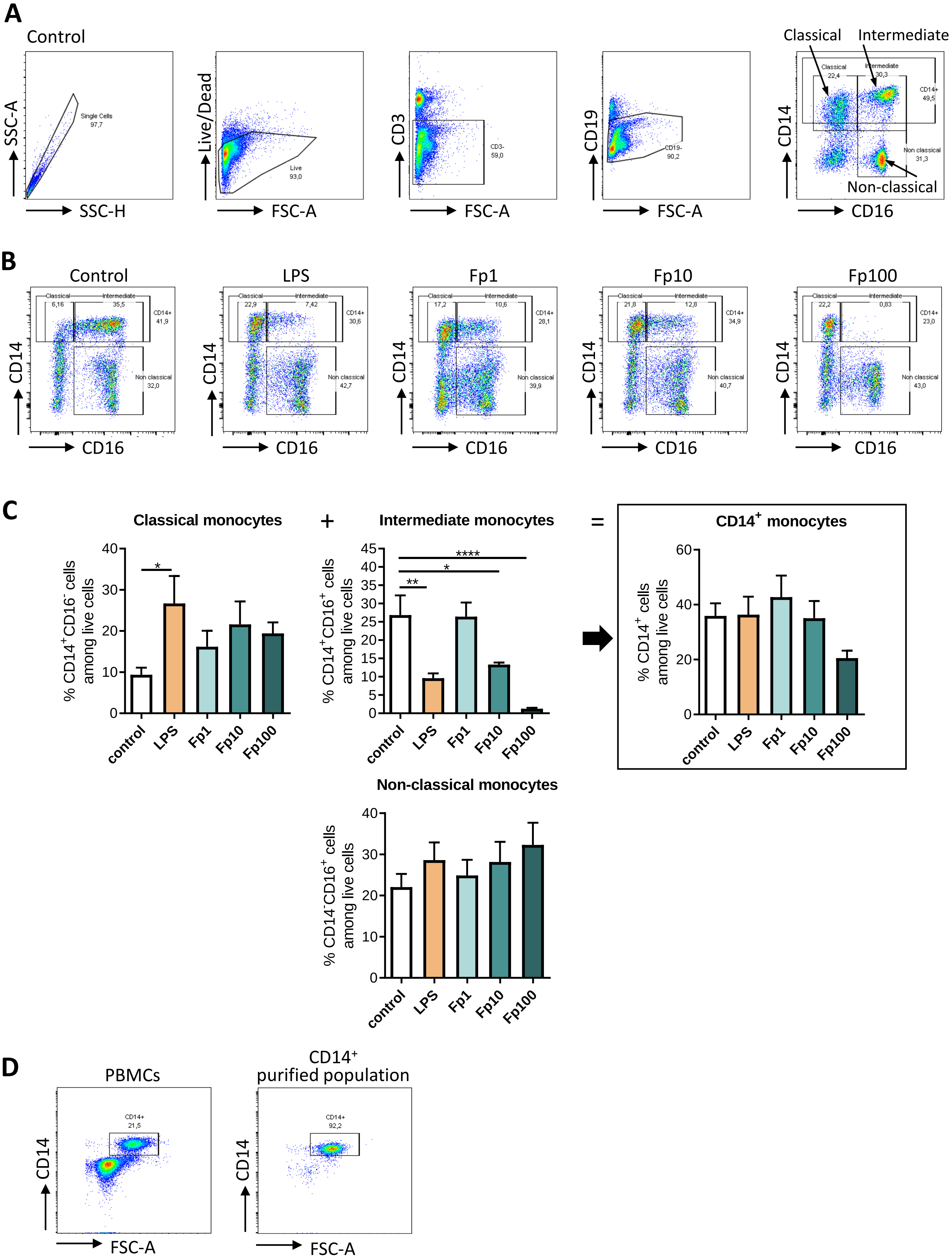
A
Control
Single Cells
97,7
SSC-A
SSC-H
Live/Dead
Live
93,0
FSC-A
CD3
CD3-
59,0
FSC-A
CD19
CD19-
90,2
FSC-A
Classical
Intermediate
Classical
22,4
Intermediate
30,3
CD14+
49,5
Non classical
31,3
Non-classical
CD14
CD16
B
Control
Classical
6,16
Intermediate
35,5
CD14+
41,9
Non classical
32,0
LPS
Classical
22,9
Intermediate
7,42
CD14+
30,6
Non classical
42,7
Fp1
Classical
17,2
Intermediate
10,6
CD14+
28,1
Non classical
39,9
Fp10
Classical
21,8
Intermediate
12,8
CD14+
34,9
Non classical
40,7
Fp100
Classical
22,2
Intermediate
0,83
CD14+
23,0
Non classical
43,0
CD14
CD16
C
Classical monocytes
% CD14+CD16- cells among live cells
+
Intermediate monocytes
% CD14+CD16+ cells among live cells
=
CD14+ monocytes
% CD14+ cells among live cells
Non-classical monocytes
% CD14-CD16+ cells among live cells
control
LPS
Fp1
Fp10
Fp100
*
**
*
****
D
PBMCs
CD14+
21,5
CD14+ purified population
CD14+
92,2
CD14
FSC-A

Suppl Fig 3

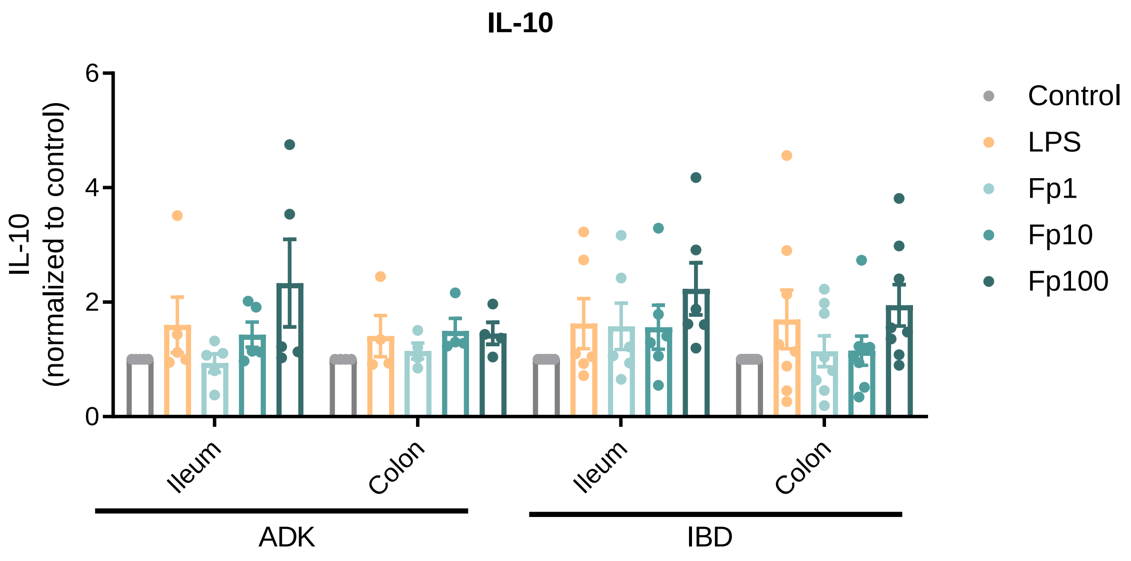
IL-10
IL-10
(normalized to control)
6
4
2
0
Ileum
Colon
Ileum
Colon
ADK
IBD
Control
LPS
Fp1
Fp10
Fp100

Suppl Fig 4

A

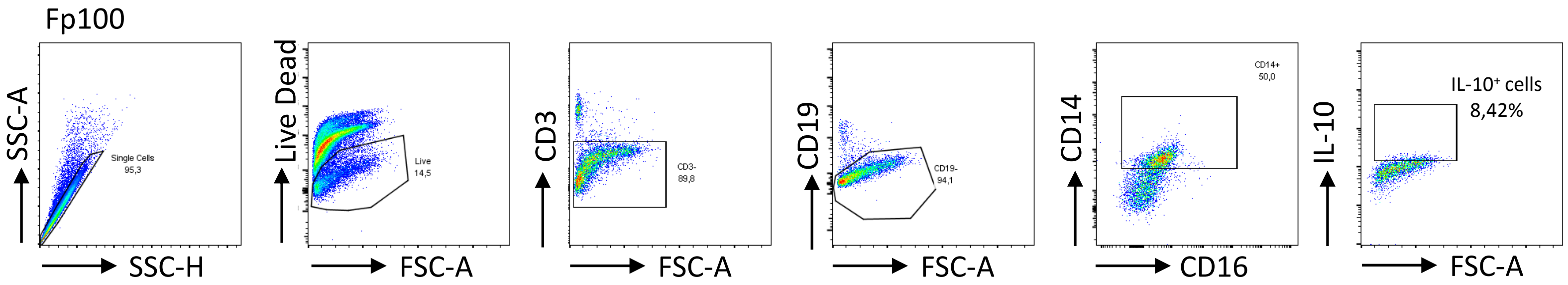

B

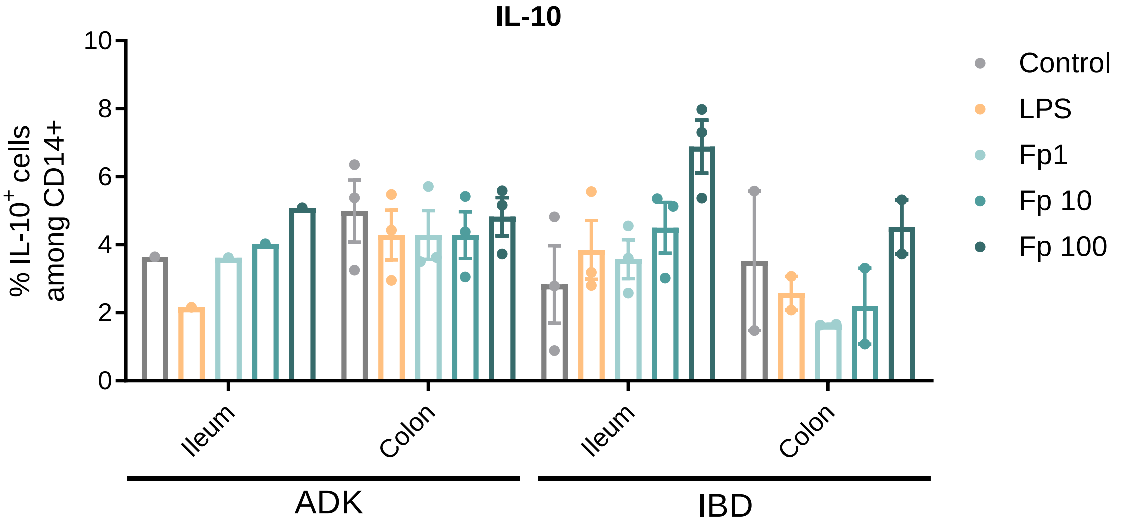

C

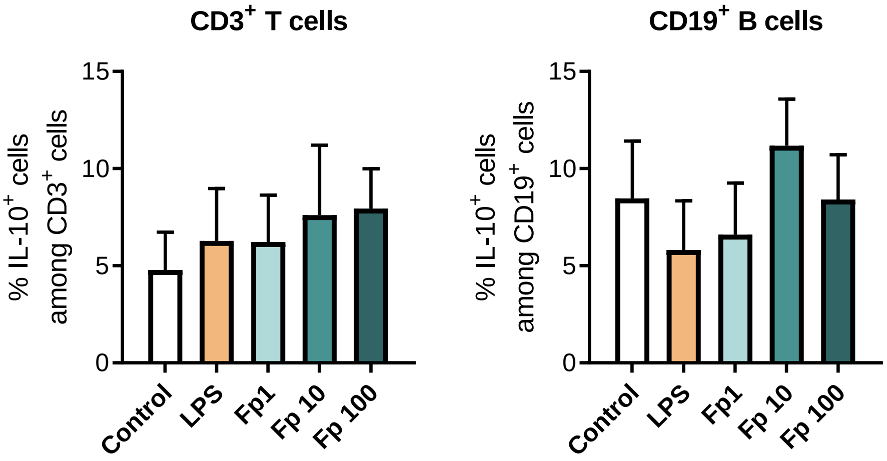

Suppl Fig 5

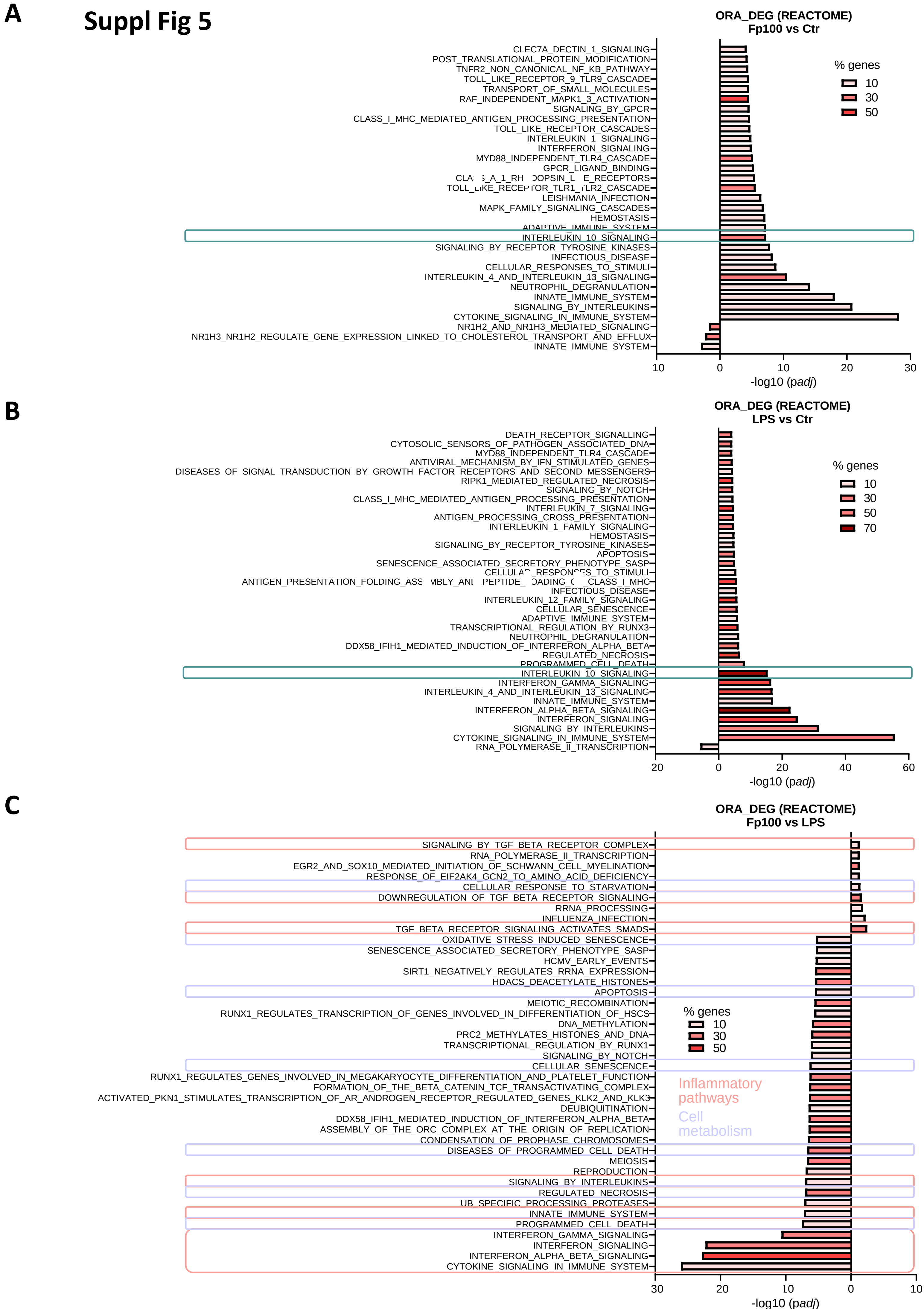

A
ORA_DEG (REACTOME)
Fp100 vs Ctr
CLEC7A_DECTIN_1_SIGNALING
POST_TRANSLATIONAL_PROTEIN_MODIFICATION
TNFR2_NON_CANONICAL_NF_KB_PATHWAY
TOLL_LIKE_RECEPTOR_9_TLR9_CASCADE
TRANSPORT_OF_SMALL_MOLECULES
RAF_INDEPENDENT_MAPK1_3_ACTIVATION
SIGNALING_BY_GPCR
CLASS_I_MHC_MEDIATED_ANTIGEN_PROCESSING_PRESENTATION
TOLL_LIKE_RECEPTOR_CASCADES
INTERLEUKIN_1_SIGNALING
INTERFERON_SIGNALING
MYD88_INDEPENDENT_TLR4_CASCADE
GPCR_LIGAND_BINDING
LEISHMANIA_INFECTION
MAPK_FAMILY_SIGNALING_CASCADES
HEMOSTASIS
INTERLEUKIN_10_SIGNALING
SIGNALING_BY_RECEPTOR_TYROSINE_KINASES
INFECTIOUS_DISEASE
CELLULAR_RESPONSES_TO_STIMULI
INTERLEUKIN_4_AND_INTERLEUKIN_13_SIGNALING
NEUTROPHIL_DEGRANULATION
INNATE_IMMUNE_SYSTEM
SIGNALING_BY_INTERLEUKINS
CYTOKINE_SIGNALING_IN_IMMUNE_SYSTEM
NR1H2_AND_NR1H3_MEDIATED_SIGNALING
NR1H3_NR1H2_REGULATE_GENE_EXPRESSION_LINKED_TO_CHOLESTEROL_TRANSPORT_AND_EFFLUX
INNATE_IMMUNE_SYSTEM
% genes
10
30
50
10
0
10
20
30
-log10 (padj)
B
ORA_DEG (REACTOME)
LPS vs Ctr
DEATH_RECEPTOR_SIGNALLING
CYTOSOLIC_SENSORS_OF_PATHOGEN_ASSOCIATED_DNA
MYD88_INDEPENDENT_TLR4_CASCADE
ANTIVIRAL_MECHANISM_BY_IFN_STIMULATED_GENES
DISEASES_OF_SIGNAL_TRANSDUCTION_BY_GROWTH_FACTOR_RECEPTORS_AND_SECOND_MESSENGERS
RIPK1_MEDIATED_REGULATED_NECROSIS
SIGNALING_BY_NOTCH
CLASS_I_MHC_MEDIATED_ANTIGEN_PROCESSING_PRESENTATION
INTERLEUKIN_7_SIGNALING
ANTIGEN_PROCESSING_CROSS_PRESENTATION
INTERLEUKIN_1_FAMILY_SIGNALING
HEMOSTASIS
SIGNALING_BY_RECEPTOR_TYROSINE_KINASES
APOPTOSIS
SENESCENCE_ASSOCIATED_SECRETORY_PHENOTYPE_SASP
INFECTIOUS_DISEASE
INTERLEUKIN_12_FAMILY_SIGNALING
CELLULAR_SENESCENCE
ADAPTIVE_IMMUNE_SYSTEM
TRANSCRIPTIONAL_REGULATION_BY_RUNX3
NEUTROPHIL_DEGRANULATION
DDX58_IFIH1_MEDIATED_INDUCTION_OF_INTERFERON_ALPHA_BETA
REGULATED_NECROSIS
INTERLEUKIN_10_SIGNALING
INTERFERON_GAMMA_SIGNALING
INTERLEUKIN_4_AND_INTERLEUKIN_13_SIGNALING
INNATE_IMMUNE_SYSTEM
INTERFERON_ALPHA_BETA_SIGNALING
INTERFERON_SIGNALING
SIGNALING_BY_INTERLEUKINS
CYTOKINE_SIGNALING_IN_IMMUNE_SYSTEM
RNA_POLYMERASE_II_TRANSCRIPTION
% genes
10
30
50
70
20
0
20
40
60
-log10 (padj)
C
ORA_DEG (REACTOME)
Fp100 vs LPS
SIGNALING_BY_TGF_BETA_RECEPTOR_COMPLEX
RNA_POLYMERASE_II_TRANSCRIPTION
EGR2_AND_SOX10_MEDIATED_INITIATION_OF_SCHWANN_CELL_MYELINATION
RESPONSE_OF_EIF2AK4_GCN2_TO_AMINO_ACID_DEFICIENCY
CELLULAR_RESPONSE_TO_STARVATION
DOWNREGULATION_OF_TGF_BETA_RECEPTOR_SIGNALING
RRNA_PROCESSING
INFLUENZA_INFECTION
TGF_BETA_RECEPTOR_SIGNALING_ACTIVATES_SMADS
OXIDATIVE_STRESS_INDUCED_SENESCENCE
SENESCENCE_ASSOCIATED_SECRETORY_PHENOTYPE_SASP
HCMV_EARLY_EVENTS
SIRT1_NEGATIVELY_REGULATES_RRNA_EXPRESSION
HDACS_DEACETYLATE_HISTONES
APOPTOSIS
MEIOTIC_RECOMBINATION
RUNX1_REGULATES_TRANSCRIPTION_OF_GENES_INVOLVED_IN_DIFFERENTIATION_OF_HSCS
DNA_METHYLATION
PRC2_METHYLATES_HISTONES_AND_DNA
TRANSCRIPTIONAL_REGULATION_BY_RUNX1
SIGNALING_BY_NOTCH
CELLULAR_SENESCENCE
RUNX1_REGULATES_GENES_INVOLVED_IN_MEGAKARYOCYTE_DIFFERENTIATION_AND_PLATELET_FUNCTION
FORMATION_OF_THE_BETA_CATENIN_TCF_TRANSACTIVATING_COMPLEX
ACTIVATED_PKN1_STIMULATES_TRANSCRIPTION_OF_AR_ANDROGEN_RECEPTOR_REGULATED_GENES_KLK2_AND_KLK3
DEUBIQUITINATION
DDX58_IFIH1_MEDIATED_INDUCTION_OF_INTERFERON_ALPHA_BETA
ASSEMBLY_OF_THE_ORC_COMPLEX_AT_THE_ORIGIN_OF_REPLICATION
CONDENSATION_OF_PROPHASE_CHROMOSOMES
DISEASES_OF_PROGRAMMED_CELL_DEATH
MEIOSIS
REPRODUCTION
SIGNALING_BY_INTERLEUKINS
REGULATED_NECROSIS
UB_SPECIFIC_PROCESSING_PROTEASES
INNATE_IMMUNE_SYSTEM
PROGRAMMED_CELL_DEATH
INTERFERON_GAMMA_SIGNALING
INTERFERON_SIGNALING
INTERFERON_ALPHA_BETA_SIGNALING
CYTOKINE_SIGNALING_IN_IMMUNE_SYSTEM
% genes
10
30
50
Inflammatory pathways
Cell metabolism
30
20
10
0
10
-log10 (padj)

Suppl Fig 6

A

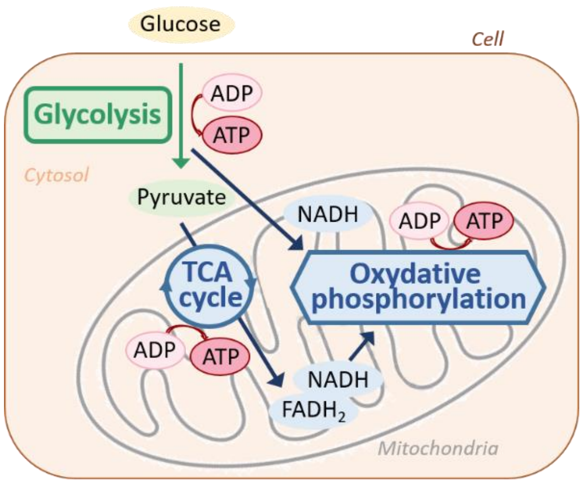

B

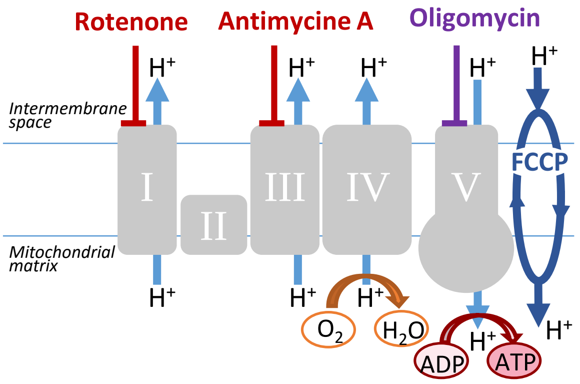

C

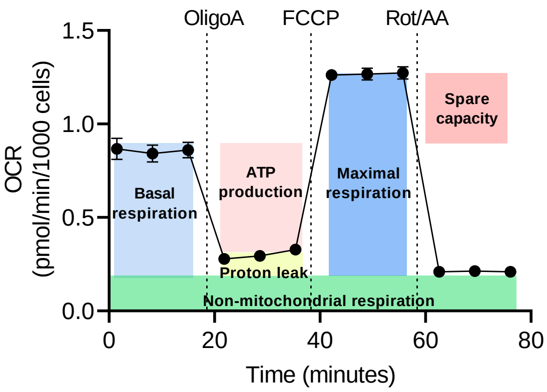

Supplement: Extended [file mmc2.pdf]
